# Access to innovation in oncology from patients’ perspectives: a qualitative systematic review

**DOI:** 10.1016/j.eclinm.2026.103892

**Published:** 2026-04-09

**Authors:** Allan Julliot-Delval, Maria Teixeira, Emmanuelle Cartron

**Affiliations:** a Siric InsiTu, INSERM, France; b Université Paris Cité, INSERM, ECEVE, F-75010, Paris, France; c Department of nursing sciences, Université Paris cité, France

**Keywords:** Oncology, Innovation, Patient perspective, Access to care, Qualitative research

## Abstract

**Background:**

Access to innovation in oncology is commonly examined from technical, organisational, or economic perspectives, yet less is known about how patients experience access along cancer care pathways. This study synthesised qualitative evidence on adult patients’ experiences of accessing innovation.

**Methods:**

We conducted a qualitative systematic review between Sept 1 and Oct 24, 2025, using the Joanna Briggs Institute meta-aggregation approach. MEDLINE, PsycINFO, Cairn.info, and OpenEdition were searched. Eligible studies reported adult patients’ experiences of access to innovation in oncology. Findings were appraised, grouped by similarity in meaning, and aggregated into synthesised findings. Confidence was assessed using the ConQual approach. This review is registered with PROSPERO, CRD420251046315.

**Findings:**

20 studies involving 306 patients were included. We identified 209 findings, grouped into 13 categories and four synthesised findings. Access to innovation was described as depending on accessible information, continuity of care, conditions supporting self-management, and the availability of spaces not centred on disease.

**Interpretation:**

Access to innovation extends beyond availability or eligibility. It emerges as a dynamic process shaped by time, care relationships, personal resources, and organisational context. Aligning innovation with patients’ lived experiences may support more equitable access.

**Funding:**

The Integrated Cancer Research Centre (SIRIC) InsiTu: Insights into Cancer: From Inflammation to Tumour.

**Research in context**

**Evidence before this study**

Access to innovation in oncology has primarily been examined from technical, organisational, or policy perspectives. Qualitative studies have described patient experiences of specific innovations, often within a single cancer type or stage of care. However, no qualitative systematic review had synthesised how adult patients experience access to innovations across different types of innovation and stages of the oncology care pathway. We therefore conducted a systematic search across four databases to identify qualitative studies reporting patient experiences of access to innovation in oncology.

**Added value of this study**

Using a meta-aggregation approach, this review synthesises qualitative evidence on patient experiences across therapeutic, organisational, educational, and sociocultural innovations. The findings show that access is not limited to availability but develops over time through experiential processesshaped by information delivery, continuity of care relationships, expectations around self-management, and access to spaces not centred on disease.

**Implications of all the available evidence**

The evidence indicates that access to innovation in oncology is shaped by relational, informational, organisational, and social conditions in addition to technical implementation factors. These findings support the development of oncology care pathways that better align innovation with patients’ lived experiences and highlight the need for further research addressing access among socially vulnerable or under-represented populations.

## Introduction

Innovation is a central component of contemporary oncology care, encompassing therapeutic and organisational developments such as targeted therapies, immunotherapies, early-phase clinical trials, and new models of care.^1^^,^^2^ Over the past decade, the development of oncology innovations has accelerated cons#iderably across multiple domains, including diagnostics, treatment, and care organisation, increasing the complexity of cancer care systems.^3^ This rapid expansion may create new challenges for patients seeking to access innovation across increasingly complex care pathways. Most existing studies focus on the effectiveness of these innovations, their dissemination, or the factors influencing their implementation.^4^^,^^5^ Access to innovation is commonly examined from economic, political, or institutional perspectives.^5^ Within this literature, innovation is often treated as an intervention to be implemented rather than an experience lived by patients.^3^^,^^5^ Studies have shown that how innovations are presented and integrated into care can influence how they are perceived and accepted.^6^

Qualitative studies in specific oncology contexts suggest that access to innovation is not a single event but a process shaped by emotional, relational, and temporal dynamics. Research on early-phase clinical trials indicates that patients’ decisions to engage with innovation are often made under conditions of urgency and uncertainty, relying on trust in clinicians and coping strategies to make sense of innovation within disrupted life trajectories.^7^ Beyond the moment of decision, studies of innovations in cancer survivorship care; such as survivorship care plans, structured follow-up programmes, or digital self-management and supportive care interventions; show that access also depends on organisational conditions, including continuity of care, adaptation over time, and integration into routine clinical practice.^8^ These findings suggest that innovation may exist without being fully accessible when patients or organisations lack the resources required for its appropriation. However, qualitative insights into access to innovation remain fragmented across specific innovations, cancer types, and stages of the care pathway, from diagnosis and active treatment to survivorship or palliative care. To address this gap, we conducted a qualitative systematic review to examine how adult patients with cancer experience access to innovation in oncology, identify barriers and facilitators shaping these experiences, and analyse how primary studies characterise innovations encountered along care pathways. In this review, innovation refers to any device, intervention, organisation, or technology described as such in primary studies and encountered within oncological care pathways.

## Methods

### Overview

This qualitative systematic review used the Joanna Briggs Institute (JBI) meta-aggregation approach, which is suited to synthesising patient-reported experiences.^9^ This pragmatic approach aggregates findings as formulated by authors of primary studies, without inductive reanalysis or theoretical interpretation, to preserve reported meanings.^9^^,^^10^

The review was conducted according to a previously published protocol^11^ and registered in PROSPERO (CRD420251046315). Methods followed the planned JBI meta-aggregation approach. One minor deviation occurred: although the Cumulative Index to Nursing and Allied Health Literature (CINAHL) was specified in the protocol, it could not be searched. All other databases were searched as planned.

In line with JBI principles, barriers, facilitators, and contextual factors were identified as described by participants and reported by study authors, without prior categorisation. Innovations were characterised using descriptions provided in the primary studies.

### Search strategy and selection criteria

Eligibility criteria were defined using the Population, Phenomenon of Interest, and Context (PICo) framework, including adult patients with cancer (Population), experiences of access to innovation (Phenomenon of Interest), and oncology care pathways (Context)in accordance with JBI recommendations.^9^ The detailed PICo framework used to define inclusion and exclusion criteria is provided in Web Appendix (Table 1). Primary qualitative studies using interviews, focus groups, observations, or ethnographic approaches were eligible. Mixed-methods studies were included when qualitative data were clearly identifiable. Quantitative studies, reviews, conceptual analyses, and editorials were excluded. No time restriction was applied. Studies published in English or French were eligible.

The search strategy followed JBI recommendations.^9^ Searches were conducted between Sept 1 and Oct 24, 2025, in MEDLINE (via PubMed), PsycINFO, OpenEdition, and Cairn.info. These databases were selected to capture qualitative research across health sciences, psychology, and the human and social sciences, including French-language qualitative studies that may not be indexed in traditional biomedical databases. Search combined controlled vocabulary and free-text terms related to oncology, innovation, and qualitative research. Strategies were reviewed by an independent third party. Strategies were reviewed by an independent third party. Full search strategies are provided in Web Appendix (Table 2) and were consistent with the published protocol.^11^ Reference lists of included studies were also screened to identify additional eligible publications.

**Fig. 1:**
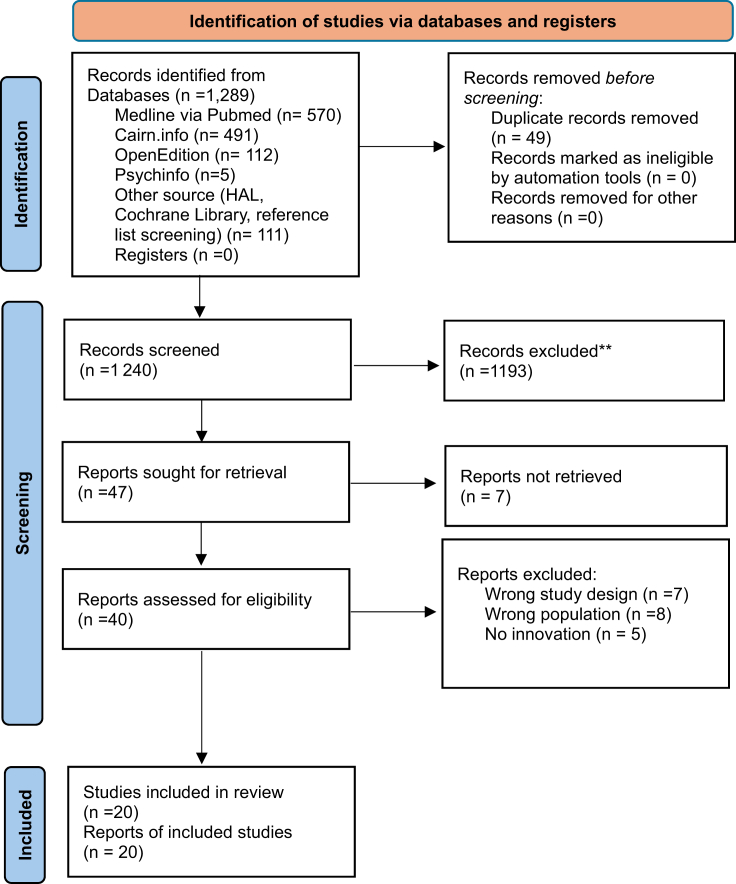
**PRISMA 2020 flow diagram for study selection.** ∗Consider, if feasible to do so, reporting the number of records identified from each database or register searched (rather than the total number across all databases/registers). ∗∗If automation tools were used, indicate how many records were excluded by a human and how many were excluded by automation tools.

Source: Page MJ et al. BMJ 2021; 372:n71. https://doi.org/10.1136/bmj.n71. This work is licenced under CC BY 4.0. To view a copy of this licence, visit https://creativecommons.org/licenses/by/4.0/.

Records were imported into Rayyan®,^12^ with duplicates removed automatically and verified manually. Titles, abstracts, and full texts were screened independently by two reviewers (AJD and EC), with disagreements resolved by discussion or adjudication by a third reviewer (MT). The selection process is reported using a PRISMA 2020 flow diagram^13^ (Fig. 1).

The research team included expertise in anthropology, nursing science, and public health. Reflexivity was maintained to consider potential disciplinary assumptions.

Patients and the public were not involved in the design, conduct, or reporting of this study. Findings will be disseminated through scientific and professional channels.

### Data analysis

Data were extracted using a standardised grid consistent with the JBI meta-aggregation approach.^9^ Extracted data included participant characteristics, study context, type of innovation, and findings related to access as formulated by study authors. Extracted data and supporting illustrations (participant quotations) are presented in the Web Appendix (Tables 3 and 4). No sex- or gender-based analyses were conducted due to heterogeneity and limited reporting in the included studies.

**Fig. 2:**
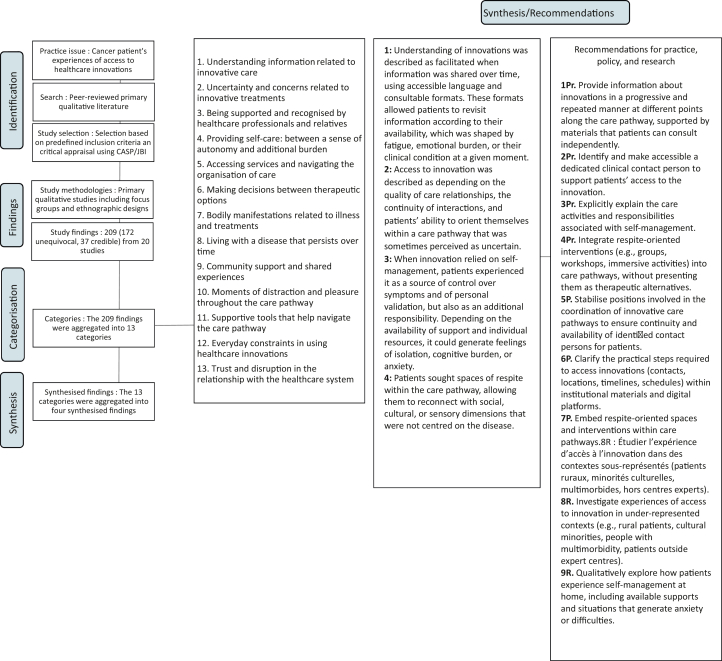
**Meta-aggregation synthesis process**.

Data synthesis followed the three-step JBI meta-aggregation process. Extracted findings and supporting illustrations were first reviewed and assigned a credibility level according to JBI guidance. Findings were appraised for credibility and classified as Unequivocal, Credible, or Not Supported; only findings rated Unequivocal or Credible were retained. Findings with similar meaning were iteratively grouped into descriptive categories. The categorisation was conducted by AJD and discussed with EC and MT to refine and validate the structure of categories and synthesised findings. Categories were then aggregated into Synthesised Findings following the JBI meta-aggregation approach. Traceability between findings, categories, and Synthesised Findings was maintained and documented (Fig. 2).

Methodological quality was assessed using the Critical Appraisal Skills Programme (CASP) Qualitative Research checklist, as specified in the protocol.^11^^,^^14^ No studies were excluded based on quality; appraisal informed the confidence assessment. Appraisal results are reported in Web Appendix (Table 5). Confidence in each Synthesised Finding was assessed using the Confidence in the Qualitative research (ConQual) approach, with results reported in the Summary of Findings table (Web Appendix Table 6).

### Ethics

This review synthesised data from published qualitative studies. Ethical approval was not required.

### Role of the funding source

The funder had no role in study design, data collection, data analysis, data interpretation, or writing of the report. The corresponding author had full access to all the data and had final responsibility for the decision to submit for publication.

## Results

Among 1289 records identified, 20 qualitative studies were included after title and abstract screening and full-text review. Studies were conducted across nine countries, most frequently in Canada (n = 4), Australia (n = 3), Denmark (n = 3), and the Netherlands (n = 3), followed by Norway (n = 2) and France (n = 2). One study each was conducted in the USA, Italy, and China. Publications spanned 2015–2025. Most studies were published in English, with two in French, and appeared in journals focussing on oncology, public health, or social sciences. Study characteristics are reported in the Web Appendix (Table 2).

A total of 209 findings were extracted: 172 Unequivocal and 37 Credible (Web Appendix Table 5). These findings were grouped into 13 categories and aggregated into four synthesised findings (Fig. 2 and Web Appendix Table 7).

The included studies used a range of qualitative designs, including descriptive and exploratory studies based on semi-structured interviews, studies informed by interpretive or constructivist approaches, ethnographic and case study designs, and qualitative components embedded within mixed-methods studies. Data collection methods included individual and group interviews, focus groups, participant or non-participant observations, and, in some studies, longitudinal or participatory approaches. Methodological characteristics are detailed in the Web Appendix (Table 3).

The 20 studies involved 306 adult patients with solid or haematological cancers (180 women, 105 men, and 21 participants for whom sex or gender was not reported). Reported ages ranged from 18 years to 94 years (mean 60.7 years). Eight studies focused on a single cancer type, nine included multiple diagnoses, and three did not specify tumour site. Two studies involved long-term cancer survivors, and three included patients receiving palliative care.

Participants were interviewed at different points along their clinical trajectories, including during active treatment (radiotherapy, chemotherapy, or home-based oral treatment), within clinical trials, after planned surgery, or during the palliative phase. In several studies, innovation emerged in pathways characterised by disease progression or the ineffectiveness of previous treatment options (n = 5). Other studies focused on individuals in remission, followed in survivorship programmes or supported during post-treatment transitions.

Studies were conducted across a range of settings, including medical or surgical oncology units, radiotherapy departments, specialised university centres, community-based services, and remote follow-up programmes. Several studies focused on home-based innovations, such as self-management programmes, digital platforms, or telemonitoring tools. Some were conducted in services with a population-level or intercultural focus, including Indigenous health centres and programmes for young cancer survivors.

In line with the secondary objective, innovations were described either as newly introduced interventions within existing services or as pilot or experimental interventions in the implementation phase. In several publications, the term “innovation” was used broadly to refer to any new intervention within a given context, without explicit distinction between technological, organisational, educational, or therapeutic dimensions. Descriptions were primarily functional, focussing on intervention characteristics, components, or context of introduction. Few studies provided a formal definition or conceptual categorisation of innovation, and only three referred to a theoretical framework.

Overall, studies demonstrated satisfactory methodological quality (Web Appendix Table 5). Study aims were clearly stated, and qualitative approaches were appropriate to the research questions. Data collection and analysis were generally rigorous, with frequent use of explicit analytical frameworks and support from participant quotations.

Identified limitations included limited contextualisation of findings, variable transparency of interpretative processes, and infrequent reporting of participant validation. Criteria for saturation were rarely described. Ethical reporting was inconsistent, and samples were often small, single-centre, or culturally homogeneous, which may limit transferability.

These considerations informed the assessment of confidence in the synthesised findings using the ConQual approach (Web Appendix Table 6). No studies were excluded based on methodological appraisal.

According to the included studies, patients’ experiences of access to innovation in oncology were described as dynamic and processual rather than event based. Access was shaped by how information was delivered and revisited over time, by the continuity and organisation of care relationships, by the conditions under which responsibility for care was delegated to patients, and by opportunities to engage in spaces not centred on disease. These four dimensions structure the synthesised findings presented below, illustrating how access to innovation develops through interactions between personal resources, care pathways, and organisational contexts.

Patients described understanding innovations as a gradual process rather than a single episode of information delivery. In some studies, patients were not only recipients of information but active information seekers, consulting multiple sources, seeking second opinions, or exploring clinical trial opportunities to better understand available options. Access was facilitated when communication used accessible language and was adapted to patients’ clinical and emotional capacity at different moments along the care pathway. Variations in clinical status, such as side effects, fatigue, pain, or emotional distress, were described as limiting the ability to process or retain information, even when it had been previously provided.

To accommodate these fluctuations, studies highlighted the importance of consultable information formats, including written materials, digital platforms, and educational videos. The possibility to reread, rewatch, or rehear information enabled patients to revisit content according to their availability and supported progressive appropriation of innovations over time.

Innovations relying on self-management were described as ambivalent. In some contexts, they fostered autonomy, control, and validation, particularly when embedded within ongoing interactions with health-care professionals that allowed feedback and adjustment over time.

In other contexts, delegation of care to the home, such as oral treatment administration, side-effect monitoring, or use of digital tools, was associated with anxiety, isolation, and cognitive burden. These experiences were shaped by the level of perceived support and ease of contact with professionals.

Access to innovation was linked to the quality and continuity of relationships with health-care professionals and to the organisation of care pathways. Patients reported greater engagement when professionals were familiar with their trajectories, attentive to concerns, and available as stable points of reference, enabling doubts to be revisited as circumstances evolved.

Conversely, disruptions in continuity, such as frequent changes in care providers, delays in care processes, or limited time for interaction were described as undermining engagement. In these situations, patients reported difficulty projecting themselves into proposed innovations, particularly when decisions were required under time pressure.

Patients described seeking spaces of respite that allowed temporary distance from disease and treatment, enabling reconnection with social, cultural, or sensory dimensions not centred on illness. Activities such as patient groups, cultural or creative practices, walking, or immersive technologies provide distraction, relief, and opportunities to share experiences outside a biomedical framework.

Beyond temporary relief, these spaces supported identity and social connection, particularly when interactions within care pathways were predominantly disease-focused and contributed to maintaining continuity with personal and social lives alongside ongoing care.

## Discussion

This qualitative synthesis shows that, from patients' perspectives, access to innovation in oncology cannot be reduced to availability, eligibility, or uptake. Instead, access emerges as a situated and evolving process of appropriation, shaped by patients' capacity to receive, understand, and integrate innovation within demanding care trajectories. This process unfolds over time and is influenced by clinical vulnerability, emotional burden, relational continuity, and care organisation. In several included studies, relatives or close social networks were described as supporting patients’ understanding of innovation, for example by helping interpret information, accompanying consultations, or assisting in decision-making. Although these references did not emerge as a distinct synthesised finding across the corpus, they highlight the relational context in which patients encounter innovation and suggest that access may also be mediated through family or social support. By synthesising qualitative evidence across different types of innovation and stages of the oncology care pathway, this review brings together experiential mechanisms that are often examined separately in the literature.

Across studies, patients' engagement with innovation was closely linked to temporal and cognitive conditions. Research in health communication shows that cognitive availability fluctuates with emotional burden, symptom severity, and uncertainty, limiting the integration of information delivered at a single point in time.^15^ Our findings extend this evidence by showing that the timing of innovation is not fixed but shifts along the clinical trajectory according to patients lived experience of illness and treatment. Understanding was often constructed retrospectively, through repeated exposure to information and opportunities to revisit content when patients felt more available. Similar processes have been described in studies of clinical trials and patient navigation, where comprehension develops progressively rather than at initial disclosure.^16^^,^^17^ Comparable dynamics have been reported in genomic medicine, where the meaning of innovation is reworked over time as information is reintegrated into lived experience.^18^ Together, these findings suggest that access depends less on the presence of innovation than on patients’ ability to situate themselves within it at a given clinical moment.

Innovation was frequently introduced at times of heightened vulnerability, such as disease progression or treatment failure, when previous therapeutic options had been exhausted.^16^ In these situations, patients described physical and emotional fragility that limited their capacity to anticipate future outcomes. Quantitative studies grounded in theories of illness uncertainty have reported associations between uncertainty, fatigue, attentional difficulties, and reduced quality of life, partly mediated by coping strategies.^19^ Our synthesis complements this evidence by showing how uncertainty operates experientially, constraining engagement with innovation by limiting patients’ ability to envisage the future. Innovation therefore enters care pathways already shaped by urgency, uncertainty, and emotional strain, rather than neutral decision-making environments.

Several studies described innovation as scarce or urgent, creating the perception that decisions had to be made rapidly.^20^ Research on early-phase clinical trials has shown that perceptions of urgency or of a “last chance” strongly influence decision-making, sometimes preceding full understanding of participation implications.^20^^,^^21^ Rather than attributing these decisions solely to individual cognitive biases, our findings suggest that they arise at the intersection of vulnerability, time pressure, and organisational constraints. In this context, acceptance of innovation was not always described as a fully deliberative choice, but as a means of maintaining continuity within disrupted care trajectories.^21^

Hope emerged as a recurrent element in these accounts, particularly when innovation was associated with advanced or aggressive disease.^21^^,^^22^ Quantitative research has shown that hope and optimism are not independently associated with survival once clinical and prognostic factors are considered.^22^ Our findings align with qualitative literature suggesting that hope functions as a coping resource that supports engagement with care in contexts of limited options, rather than as an indicator of misunderstanding or denial.^21^

Innovations relying on self-management further illustrated this ambivalence. Autonomy was experienced as empowering when embedded within sustained relational and organisational support, whereas delegation of care to the home generated anxiety, isolation, and cognitive burden when feedback was limited or difficult to access.^23^ Quantitative evidence indicates that organisational interventions such as patient navigation improve treatment initiation, adherence, and patient satisfaction, particularly among disadvantaged populations.^17^ Our synthesis suggests that these effects may be mediated by patients’ ability to orient themselves within coherent and predictable care pathways, highlighting experiential mechanisms through which organisational continuity supports access.^24^

Non-technical innovations, including creative, collective, or immersive interventions, occupied a distinct place in patients’ narratives. These interventions were rarely framed as innovations per se, but rather as spaces of respite where illness and treatment temporarily ceased to structure everyday life.^25^ Their acceptability depended less on novelty than on the experiences they enabled, including social connection, identity continuity, and relief from treatment-related burden.^26^ Such interventions were more frequently described in supportive or palliative care contexts, where quality of life and long-term adjustment are central concerns.^27^ Compared with biomedical innovations, they generated less uncertainty, partly because they were perceived as having no direct impact on disease progression.^24^^,^^25^ By offering moments of respite, they were described as supporting sustained engagement with care over time.^28^

Across the qualitative literature, innovation was rarely defined explicitly. The term was often used to denote novelty or local implementation, without clarifying its defining characteristics.^29^ Conceptual frameworks were more frequently applied to evaluate intervention effects than to interrogate innovation as a concept. This variability may contribute to the diversity of patient-reported experiences of access observed across studies. Our synthesis highlights the importance of situating innovation within care pathways and examining how it is introduced, framed, and experienced, rather than treating it as a self-evident category.^29^

By integrating patient experiences across heterogeneous innovations and clinical contexts, this review provides a cross-cutting understanding of access as an experiential process. It suggests that the design and implementation of person-centred innovations require attention not only to technical effectiveness, but also to temporal, relational, and organisational conditions shaping patients' capacity to engage. Aligning innovative strategies with patients lived experiences may help reduce inequities in access and support more responsive oncology care pathways. These findings resonate with conceptual frameworks of patient-centred access to healthcare, which emphasise the interaction between health system characteristics and patients’ capacities to engage with care.^30^ Based on the synthesised findings, practice, policy, and research-oriented recommendations were developed to support patient-centred access to innovation. These recommendations are presented in Web Appendix Fig. 2 and should be interpreted as guidance informed by qualitative evidence rather than prescriptive standards.

This qualitative systematic review has several strengths. To our knowledge, it is the first qualitative synthesis to examine patient experiences of access to innovation across oncology care pathways. The review followed a previously published protocol and used the Joanna Briggs Institute meta-aggregation approach,^9^ ensuring transparency and traceability between primary findings, credibility appraisal, and confidence in the synthesised findings. Study selection relied on independent double screening in line with PRISMA recommendations.

A further strength lies in the diversity of innovations examined, including therapeutic, organisational, educational, and sociocultural or supportive approaches. This breadth enabled identification of conditions shaping access across care settings and stages of the oncology care pathway without reducing patient experience to a single dimension of innovation.

Several limitations should be acknowledged. First, inclusion was restricted to studies published in English or French, which may have limited representation of experiences from some cultural or health system contexts. Second, although CINAHL was specified in the protocol, it could not be searched due to access constraints, and some qualitative studies published in nursing or allied health journals may therefore have been missed.

This qualitative synthesis highlights the transferability of experiential conditions shaping access to innovation in oncology. Across diverse clinical contexts, access was embedded within care trajectories often marked by fatigue, uncertainty, and organisational constraints. The consistency of these patterns across settings supports the relevance of the findings beyond individual innovations or care contexts.

From patients’ perspectives, access to innovation cannot be reduced to availability or formal eligibility. Instead, it develops through a process of appropriation shaped by how information is conveyed over time, the continuity and organisation of care relationships, and opportunities to engage in spaces not solely centred on illness. Acceptability emerges at the intersection between proposed innovations and the clinical, emotional, and organisational conditions present at the time of their introduction.

By describing these dynamics, this review provides pragmatic reference points for clinical practice and care organisation. It supports a view of access not as a discrete event, but as a progressive alignment between innovation and an evolving care trajectory, clarifying the conditions under which patients are able to consider, understand, and engage with innovation over time.

Across the primary studies, innovation was most often approached through intervention characteristics, with limited conceptual reflection on innovation as an experiential process. Greater conceptual clarity may help better capture patient expectations and support the design of approaches centred on lived experience rather than disease alone.

Finally, the limited attention paid to social determinants of access in the primary literature should be acknowledged. Socioeconomic conditions, precarious living situations, and minority or migrant backgrounds were rarely examined, despite their potential influence on experiences of access. Financial and material constraints, such as treatment costs, transport, or access to digital resources, were also only marginally addressed in the available qualitative literature, although they may substantially shape patients’ ability to engage with innovative care pathways.^31^

## Contributors

AJD conceptualised the study and developed the protocol, with input from MT and EC. AJD and EC conducted the literature search, screened studies, and extracted the data. AJD led the data synthesis and drafted the manuscript. MT and EC contributed to data interpretation and critically revised the manuscript. AJD and EC verified the underlying data.

All authors had full access to all the data in the study and had final responsibility for the decision to submit for publication. All authors read and approved the final version of the manuscript.

## Data sharing statement

Data extracted from included studies and the analytic materials supporting the findings (including the extraction grid and coding/aggregation tables) will be made available upon reasonable request to the corresponding author. No individual participant data were collected for this study.

## Declaration of interests

Allan Julliot-Delval, Maria Teixeira, and Emmanuelle Cartron are affiliated with the SIRIC InsiTu. The authors declare no other competing interests.

## Appendix A Supplementary data

Supplementary Material

Protocol
