# Supplementary material for: Access to innovation in oncology from patients’ perspectives: a qualitative systematic review
Source: eClinicalMedicine. 2026 Apr 9;94:103892. doi: 10.1016/j.eclinm.2026.103892 (PMC13091739; doi:10.1016/j.eclinm.2026.103892)
Supplement: Supplementary Material [file mmc1.pdf]

Web Appendix

Table of Contents

Web Appendix Table 1. Population, Phenomenon of Interest, and Context (PICo) framework used to define inclusion and exclusion criteria

Web Appendix Table 2. Full search strategies used across databases

Web Appendix Table 3. Characteristics of included qualitative studies

Web Appendix Table 4. List of extracted qualitative findings by study

Web Appendix Table 5. Critical Appraisal Skills Programme (CASP) quality appraisal of included studies

Web Appendix Table 6. Summary of Findings and Confidence in the Qualitative research (ConQual) assessments

Web Appendix Figure 1. Visual representation of synthesised findings

Web Appendix Figure 2. Practice, policy, and research recommendations derived from the synthesis

Web Appendix Table 1: Population, Phenomenon of Interest, and Context (PICo) framework used to define inclusion and exclusion criteria

PICo component	Definition
**Population (P)**	Adult patients (≥18 years) with cancer, regardless of histological type, disease stage, or therapeutic trajectory. Studies focusing exclusively on relatives, caregivers, or health-care professionals, or those not allowing separate extraction of patient-derived data, were excluded. Sociodemographic and medical characteristics (age, sex, pathology) were collected for descriptive purposes.
**Phenomenon of Interest (I)**	Experiences of access to innovation in oncology.
**Context (Co)**	Oncology cares in any setting (hospital, outpatient, or community-based), regardless of institution type (public, private, or voluntary sector) or geographical and cultural context.

Web Appendix Table 2: Full search strategies used across databases

Database	Search strategy
**PubMed**	#1 MESH DESCRIPTOR: Innovation EXPLODE ALL TREES #2 innovati*[tiab] #3 "health care reform"[tiab] #4 "new model"[tiab] #5 "organizational change"[tiab] #6 "emerging practice"[tiab] #7 #1 OR #2 OR #3 OR #4 OR #5 OR #6 #8 MESH DESCRIPTOR: Neoplasms EXPLODE ALL TREES #9 cancer[tiab] #10 oncology[tiab] #11 "cancer patient*"[tiab] #12 #8 OR #9 OR #10 OR #11 #13 MESH DESCRIPTOR: Qualitative Research EXPLODE ALL TREES #14 MESH DESCRIPTOR: Interviews as Topic #15 MESH DESCRIPTOR: Focus Groups #16 MESH DESCRIPTOR: Narration #17 qualitative study[tiab] #18 interview*[tiab] #19 ethnographic[tiab] #20 "grounded theory"[tiab] #21 phenomenological[tiab] #22 thematic analysis[tiab] #23 "lived experience"[tiab] #24 #13 OR #14 OR #15 OR #16 OR #17 OR #18 OR #19 OR #20 OR #21 OR #22 OR #23 #25 #7 AND #12 AND #24 #26 english[lang] OR french[lang] #27 caregiver*[tiab] OR "health professional*"[tiab] OR nurse*[tiab] OR physician*[tiab] OR oncologist*[tiab] OR clinician*[tiab] #28 #25 AND #26 NOT #27
**PsycINFO**	(TI,AB(innovation)) AND<br>(TI,AB("cancer patients" OR "oncology patients" OR "patients with cancer")) AND<br>(TI,AB(experiences OR perceptions OR attitudes OR views))<br>Limits: Peer-reviewed journal articles; English OR French language
**Cairn.info**	Keywords: ("expérience patient") AND ("innovation") AND ("cancer" OR "oncologie")<br>Filters: Journal articles, French OR English language
**OpenEdition**	Keywords: ("expérience patient") AND ("innovation") AND ("cancer" OR "oncologie")<br>Filters: Journal articles, French OR English language

Web Appendix Table 3: Characteristics of included qualitative studies

Article ID	Firth author/year	Title	Country	Language of publication	Phenomenon of interest	Study design and methodology	Participants, sample
**Art-1**	Myren et al, 2020	Openness to new perspectives created by patient participation at the morbidity and mortality meeting	Netherlands	English	To explore the feasibility and modalities of patient participation in morbidity and mortality meetings.	Qualitative study (semi-structured interviews).	N = 8; women; age not reported; gynaecological cancers (vulva, ovary, cervix, endometrium)
**ART-2**	Cusimano et al, 2020	Supported self-management as a model for end-of-life care in the setting of malignant bowel obstruction: A qualitative study	Canada	English	To understand the experiences of women with advanced gynaecological cancer receiving an innovative home-based follow-up.	Descriptive qualitative study (semi-structured interviews; face-to-face or telephone).	N = 15; women; mean age 66 years; gynaecological cancers with malignant bowel obstruction (MBO); advanced stage; palliative care.
**ART-3**	Matthew et al, 2025	Implementation of a sexual health clinic in an oncology setting: patient and provider perspectives	Canada	English	To explore factors influencing the implementation of a Sexual Health Clinic (SHC) in a cancer centre.	Qualitative study (virtual semi-structured interviews).	N = 6; 4 men, 2 women; age not reported; mixed cancer types (prostate, gynaecological, testicular, bladder, renal, head and neck).
**ART-4**	Ranes et al, 2022	Determinants of exercise adherence and maintenance for cancer survivors: Implementation of a community-based group exercise program. A qualitative feasibility study	Norway	English	To explore how cancer survivors experience a community-based group exercise programme.	Descriptive qualitative study (focus groups).	N = 14; 8 men, 6 women; age 50–77 years (mean 65); mixed cancer types among survivors (prostate, breast, rectum, fallopian tube).
**ART-5**	Kristensen et al, 2024	Exploring patient-reported barriers to participating in proton therapy clinical trials	Denmark	English	To identify perceived barriers to participation in a randomised trial of proton therapy and their influence on the decision to participate.	Qualitative study informed by Interpretive Description (semi-structured interviews).	N = 14; 8 men, 6 women; age 53–75 years (mean 66); head and neck cancers; patients who declined a proton therapy RCT.
**ART-6**	Ala-Leppilampi et al, 2020	Cancer patients' experiences with immune checkpoint modulators: A qualitative study	Canada	English	To explore the experiences of patients receiving immune checkpoint inhibitors and their impact on everyday life.	Qualitative study with an interpretive aim (individual interviews and focus groups; face-to-face or telephone).	N = 37; 13 women, 24 men; median age 60 years (range 24–85); advanced incurable cancers (melanoma n = 18, sarcoma n = 4, gynaecological n = 4, gastrointestinal n = 3, genitourinary n = 3, endocrine n = 2, head and neck n = 2, lung n = 1).
**ART-7**	Wang et al, 2022	Preferences of oral nutritional supplement therapy among postoperative patients with gastric cancer: Attributes development for a discrete choice experiment	China	English	To explore the preferences of patients who have undergone surgery for gastric cancer regarding oral nutritional supplements.	Preparatory qualitative study embedded within a mixed-methods design (semi-structured interviews and focus groups; face-to-face or telephone).	N = 34; interviews: 15 (7 men, 8 women; mean age 56 years); focus groups: 19 (7 men, 12 women; mean age 55 years); patients who underwent surgery for gastric cancer.
**ART-8**	Piil et al, 2023	The development of medical infographics to raise symptom awareness and promote communication to patients with cancer: A co-creation study	Denmark	English	To develop and evaluate infographics aimed at improving symptom communication in oncology.	Qualitative study using think-aloud interviews within a participatory design approach.	N = 15; adults receiving cancer treatment (curative or palliative); age 31–60 years; cancer types not reported.
**ART-9**	Garrett et al, 2018	Approaches to decision-making among late-stage melanoma patients: a multifactorial investigation	USA	English	To explore therapeutic decision-making processes among patients with advanced melanoma.	Multisite ethnographic study (clinical observations and longitudinal semi-structured interviews).	N = 13; 7 men, 6 women; age 30–80 years (mean 59); metastatic melanoma; longitudinal observations (n = 26) and interviews (n = 29).
**ART-10**	Oelschlägel et al, 2024	Implementation of remote home care: assessment guided by the RE-AIM framework.	Norway	English	To evaluate the implementation and sustainability of a home-based remote monitoring intervention.	Longitudinal qualitative study.	N = 11; 5 women, 6 men; mean age 66 years (range 30–94); palliative phase; cancer type not reported.
**ART-11**	Hammer et al, 2024	Feasibility and acceptability of active book clubs in cancer survivors – anexplorative investigation	Denmark	English	To explore the feasibility and acceptability of an intervention combining audiobooks, walking, and book clubs among cancer survivors.	Exploratory descriptive qualitative study (interviews).	N = 17; 71% women; median age 58 years; cancer types represented: prostate (29%), breast (24%), cervical (12%), lung, colon, lymphoma, myeloma, uterus, and ovary (6% each).
**ART-12**	Burrai et al, 2017	Immersive Virtual Reality Experience of Patients with Cancer During Intravenous Antiblastic Therapy: A Qualitative Study	Italy	English	To explore the experiences of patients using immersive virtual reality during antineoplastic treatment sessions.	Descriptive qualitative study (individual semi-structured interviews).	N = 24; mean age 63 years; predominantly Italian nationality; cancer types: breast (n = 12, including 5 stage IV), rectum (n = 2), colon (n = 2), skin (n = 2), pancreas (n = 1), testis (n = 1), prostate (n = 1), stomach (n = 1), oesophagus (n = 1), ovary (n = 1).

**ART-13**	Sier et al, 2024	Views and experiences of healthcare professionals and patients on the implementation of a 23-hour accelerated enhanced recovery programme: a mixed-method study	The Netherlands	English	To explore perceptions and experiences related to the implementation of the CHASE protocol.	Qualitative study embedded within a mixed-methods design (focus groups and post-intervention semi-structured interviews).	N = 11; 5 men, 6 women; median age 72 years; colon cancer.
**ART-14**	Wolters-Zwolle et al, 2022	Patients' experiences with an audio-visual intervention, the use of a tailored explanimation video in patients with bladder cancer.	the Netherlands	English	To explore how patients with bladder cancer perceive the use of an explanatory video for information provision and therapeutic decision-making.	Exploratory descriptive qualitative study (individual semi-structured interviews and field notes).	N = 12; 8 men, 4 women; mean age 70 years; bladder cancer.
**ART-15**	Hou et al, 2024	Co-Designing Priority Components of an mHealth Intervention to Enhance Follow-Up Care in Young Adult Survivors of Childhood Cancer and Health Care Providers: Qualitative Descriptive Study	Canada	English	To identify, through a co-design approach, priority components of an mHealth intervention for young adult survivors of childhood cancer.	Descriptive qualitative study (semi-structured interviews).	N = 22; 95% women; mean age 29 years (SD 4.78); cancer types: leukaemia (50%), lymphoma (27%), solid tumours (23%).
**ART-16**	Taylor et al, 2021	"The support has been brilliant": experiences of Aboriginal and Torres Strait Islander patients attending two high performing cancer services.	Australia	English	To explore the experiences of Indigenous patients within oncology services integrating innovative organisational and cultural approaches.	Qualitative case study (semi-structured interviews).	N = 5; 4 men, 1 woman; cancer type not reported.
**ART-17**	Marmorat, 2018	Expériences médicamenteuses et expériences du cancer	France	French	To identify the experiential knowledge mobilised by patients receiving oral anticancer therapies to manage their illness and treatment.	Constructivist qualitative study (individual semi-structured interviews).	N = 16; 6 men, 10 women; mean age 60 years; cancer types: haematological malignancies (n = 7), melanoma (n = 6), solid tumours (kidney, lung, breast; n = 3).
**ART-18**	Derbez, 2015	Devenir sujet de recherche	France	French	To understand how some patients integrate clinical research into a life project aimed at “buying time”.	Ethnographic case study (participant observation and repeated interviews).	N = 11; 7 women, 4 men; metastatic or locally advanced cancers (types not reported).
**ART-19**	Flore, 2023	Entanglements and imagined futures: The subject(s) of precision in oncology.	Australia	English	To explore the experiences of patients with metastatic lung cancer receiving targeted therapy or immunotherapy.	Descriptive and interpretive qualitative study (semi-structured interviews).	N = 20; 13 women, 7 men; age 34–83 years (median 62); metastatic non-small cell lung cancer (stage IV); diverse cultural backgrounds.
**ART-20**	Halkett et al, 2020	Supporting and preparing patients for radiotherapy: Patients’ and radiation therapists’ perspectives on their one-to-one consultations	Australia	English	To explore patients’ perceptions of the RT Prepare intervention.	Descriptive qualitative study (semi-structured interviews).	N = 21; women; mean age 58 years; breast cancer.

Web Appendix Table 4: List of extracted qualitative findings by study

Study ID	Finding	Illustration	Credibility
ART-1	**A1.1** Participation enables patients to understand team-based work, thereby enhancing their confidence.	*"That things are discussed in this manne and that you are taken seriously, in your whole story". Patient 8*	C
	**A1.2** Participation is perceived as helping patients better understand adverse effects and reducing emotional burden.	*"We wanted to see what happened, because we experienced a lot of stress and therefore we have missed some information and what exactly happened." (Patient 8)*	U
	**A1.3** Adaptation of medical language is necessary to ensure patient understanding.	*"And yes, it was all very clear…"*	C
	**A1.4** Innovations co-developed with patients are perceived as transferable and empowering.	*"If I can contribute something that will provide someone else with better treatment, or something similar, I would like to be involved in that."*	C
ART-2	**A2.1** The Self-Management Support (SMS) programme is perceived as a source of autonomy, safety, and symptom control.	*""I've learned that bowel obstruction is part and parcel of the end-stage of ovarian cancer. But I've also learned that it can be managed. You can't make the disease go away. But you can make it slightly better." (ID12)*	U
	**A2.2** Access to the programme provides emotional security and support that alleviate fear and loneliness.	*"I'm not worried about it like before. It helps the mind most because you know people are there to help you." (ID10) …"*	U
	**A2.3** The innovation improves understanding of end-of-life care.	*"If I had to call in with symptoms, they have me on record as part of the program. They know my history and they can help."*	C
	**A2.4** The innovation is experienced as a learning process that enables patients to regain a sense of control.	*"By getting into this program, I now know who to reach out to."*	C
	**A2.5** Relational continuity transforms fear into trust.	*"I can reach the nurses easily. If I wanted to contact them, I'd call and they'll respond in an hour. I'm not jumping into deep water with nothing." (ID12)*	C
	**A2.6** Persistent fear of disease progression and death remains despite the support provided.	*"I'm always afraid that this is going to be the kiss of death."*	U
	**A2.7** Palliative care is associated with a poor prognosis.	*"To me it's always fixed in my head that it's the end."*	U
	**A2.8** The SMS programme provides reassuring and accessible support, strengthening patients' sense of security.	*"They're always there if I have a problem."*	C
ART-3	**A3.1** Sexual health is not initiated by healthcare professionals; patients must actively seek information.	*"It wasn't something that was discussed with me…"*	U
	**A3.2** Perceived lack of consultation time hinders discussion of sexuality.	*"I felt like it was too long… you don't want to take up other people's time."*	U
	**A3.3** Patients wish Sexual Health Care (SHC) to be introduced at an appropriate time (after initial treatment).	*"After the dust has settled… after their first treatment."*	U
	**A3.4** Visual materials facilitate the introduction of SHC.	*"Pamphlets, signs, posters should be used."*	U
ART-4	**A4.1** The programme provides improved well-being and restored autonomy, helping participants feel physically capable.	*"Those four months have lifted me… now I can stand on my own two feet."*	U
	**A4.2** The group is experienced as a supportive and motivating community.	*"You found a community… it became the highlight of the week!"*	U
	**A4.3** Group participation helps shift the focus toward health rather than illness	*"I didn't want to be reminded… but then I found this amazing group!"*	C
	**A4.4** Conventional gyms are perceived as impersonal and unsuitable after cancer.	*"Fitness centers… impersonal and lonely environment."*	U
	**A4.5** The programme's fixed structure and regular sessions foster commitment and a sense of responsibility toward the group.	*"A set appointment… because we're a group."*	U
	**A4.6** Exercise is perceived as enhancing well-being and motivating continued engagement.	*"I just have to keep going whether I want to or not."*	C
ART-5	**A5.1** The diagnosis is experienced as a major shock.	*"It was indeed a big shock…"*	U
	**A5.2** Immediate priority is survival rather than participation in a randomised controlled trial (RCT).	*"My primary focus is on surviving."*	U
	**A5.3** Emotional burden hinders understanding of information.	*"The information went over my head…"*	U
	**A5.4** Patients experience information overload at the end of consultations.	*"The amount of information is overwhelming…"*	U
	**A5.5** Radiotherapy is presented as a difficult treatment, reinforcing emotional burden.	*"It would be a tough game."*	U
	**A5.6** Proximity to the treatment centre facilitates access.	*"It only takes me 5–10 minutes…"*	U
	**A5.7** Home environment and family support are essential.	*"My greatest source of strength lies in my home…"*	U
	**A5.8** Lack of evidence regarding proton therapy generates uncertainty.	*"I questioned its advantages…"*	U
	**A5.9** Randomisation is experienced as a loss of control.	*"The element of chance, particularly the drawing of lots, contributed to my feeling of uncertainty"*	C
	**A5.10** Waiting before treatment is difficult to accept.	*"I didn't want to wait another week…"*	U
ART-6	**A6.1** Profound and indescribable fatigue.	*"Deep tired feeling…"*	U
	**A6.2** Perceived physical decline and loss of former identity.	*"I was once such a go getter."*	U
	**A6.3** Intense itching.	*"Itch… draw blood."*	U
	**A6.4** Generalised pain and reduced mobility.	*"Everything aches."*	U
	**A6.5** Hypervigilance regarding side effects.	*"Waiting for the next shoe to drop."*	U
	**A6.6** Anxiety related to new treatments.	*"All of this stuff is so new…"*	U
	**A6.7** Feelings of abandonment.	*"very low point" as he felt "orphaned" and was left "floating"*	C
	**A6.8** Difficulty attributing symptoms.	*"Hard to tell what the med is doing."*	U
	**A6.9** Side effects perceived as signs of treatment effectiveness.	*"Good sign the drug is working."*	U
	**A6.10** Hope persists despite the burden of side effects.	*"Nothing to lose… hope."*	U
	**A6.11** Patients adapt their daily activities to illness- and treatment-related physical limitations in order to "keep living".	*"Oh no, I just carried on. I had to cut back on some of the physical activities for sure, but other than that you just make do. You carry on."*	C
	**A6.12** Essential support from healthcare professionals.	*"Doctors were brilliant."*	U
	**A6.13** Crucial family and social support.	*"Friends rallied around…"*	U
	**A6.14** Treatment is perceived as a therapeutic breakthrough, as it mobilises the immune system rather than directly attacking the cancer.	*"It's a miracle the way it works because it's a whole new breakthrough; it's not attacking the cancer; it's energizing my own immune system to do it."*	U
ART-7	**A7.1** The taste of oral nutritional supplements (ONS) is often perceived as unpleasant.	*"It tasted like milk powder… like rust… the taste is not particularly good". "The more you drink it, the worse it tastes… you can only bite the bullet and drink it."*	U
	**A7.2** Availability of multiple flavours would promote adherence.	*"If there were multiple flavors… I like sweet ones, and I would continue to drink them."*	U
	**A7.3** Choice of product format is influenced by ease of storage.	*"I chose powder… bottled supplements are not easy to store after opening."*	U
	**A7.4** Liquid formulations facilitate calculation of nutritional intake.	*"I'm drinking a liquid nutrient because the powder is not easy to count calories."*	U
	**A7.5** Gastrointestinal side effects (nausea, diarrhoea, vomiting) hinder adherence.	*"I felt nauseous every time…"<br>"If I had diarrhea as soon as I drank it, I did not want to drink it."*	U
	**A7.6** Patients develop strategies to reduce discomfort when taking ONS.	*"I adjusted the method to drink slowly… I gradually got used to it."*	U
	**A7.7** Cost influences the ability to continue ONS treatment.	*"If the price is lower, I can continue…" "Insurance helps reimburse part of it."*	U
	**A7.8** Hospitals are perceived as the best option for obtaining ONS considered "safe".	*"I think it's safer to be prescribed by the physician in the hospital."*	U
	**A7.9** Online purchases are cheaper but perceived as less secure.	*"My child bought online… it was cheaper than the hospital."*	U
	**A7.10** Preparation of ONS is often perceived as burdensome.	*"I have to stir and mix it… it is inconvenient."<br>"Two scoops are thick… not easy to calculate energy."*	U
	**A7.11** Some patients find ONS easy to use.	*"I think this nutrient is very convenient to use… like drinking milk powder."*	U
	**A7.12** Physician recommendation leads to strong adherence.	*"If he wants me to drink it… I have to drink it obediently as a medicine."*	U
	**A7.13** Lack of dietary guidance makes ONS use difficult.	*"If I don't have guidance of a dietitian at home, I don't know where to get this knowledge."*	U
	**A7.14** Nursing advice improves tolerance of oral nutritional supplements.	*"The nurse told me to drink slowly… no discomfort."*	U
	**A7.15** Educational sessions are highly requested.	*"If there are rehabilitation lectures… I would be willing to listen."*	U

	**A7.16** The nutrition-focused WeChat group is perceived as helping to avoid mistakes and better understand recommendations.	*"The nutrition WeChat group helped me avoid many detours."*	U
	**A7.17** Patients request simple guides (PDFs, e-books).	*"Better to have books in electronic version, easy to understand."*	U
	**A7.18** Patients consider individual consultations to be the best way to receive personalised information but perceive them as inaccessible due to limited human resources.	*"One-on-one consultation is best. ... After all, human resources are limited, and the number of our patients is relatively large."*	U
	**A7.19** Patients request regular follow-up (monthly or bimonthly), as it reinforces a sense of being supported and cared for.	*"Preferably once a month… I feel cared for."*	U
	**A7.20** Very frequent follow-up, particularly early in the care pathway, is considered unnecessary and of no added benefit.	*"Follow-up in the first two weeks is unnecessary."*	U
	**A7.21** Limited digital literacy among some patients leads to a preference for direct channels (telephone), perceived as more accessible than technological innovations.	*"I can't see clearly, and high-tech things such as the Internet can be difficult for me."*	U
	**A7.22** Face-to-face encounters are perceived as more reassuring and allow healthcare professionals to consider the person holistically.	*"Face-to-face follow-up is more beneficial… can see the whole person."*	U
	**A7.23** Patients want educational videos delivered via WeChat.	*"If the hospital pushes related videos… I'm willing even if a fee is charged."*	U
	**A7.24** Patients describe an ideal follow-up duration of 1 to 6 months, with several regular contacts, fostering a sense of continuity of care.	*"Communicate once a month or two… four or five times is good."*	U
	**A7.25** Videos reduce the need for repeated follow-up.	*"After the teaching video… there was no problem if I followed instructions."*	U
	**A7.26** Family support facilitates acceptance of ONS by reiterating and clarifying information provided by healthcare professionals.	*"At first, I was resistant… my wife and daughter explained… I accepted it."*	U
	**A7.27** Discontinuation of ONS occurs when patients do not perceive tangible benefits (e.g., weight maintenance).	*"I stopped taking the nutritional powder for half a year. I mainly felt that there was no nutrition and the weight was not maintained."*	U
	**A7.28** Family support encourages continued ONS use.	*"My child wants me to continue… better for me."*	U
	**A7.29** Dietitian reminders promote long-term adherence.	*"The dietitian reminded and persuaded me… I insisted for more than three months."*	U
**ART-8**	**A8.1** Infographic colours elicit emotions and personal associations that influence interpretation.	*"Purple is an emotional colour. The intestines are very sensitive too." (P3)* *"I think the colours are beautiful… they're happy… they ask you to get started on a new life." (P13)*	U
	**A8.2** Some images are difficult to interpret (shapes, symbols, colours), hindering understanding.	*"I find the green one confusing… I can't figure out where the symbols are or how to position myself." (P4)*	U
	**A8.3** Some images evoke hope and a more positive future.	*"I don't know if it's supposed to look like a butterfly… something bright and good in the future." (P13)*	U
	**A8.4** Fatigue, limited concentration, and visual size influence the ability to understand infographics.	*"When you have cancer, you might have a hard time concentrating… you can't read at all… if you have to stand up to read more, you won't get the message." (P3)*	U
	**A8.5** Excessive text or information reduces message impact and readability.	*"The worst thing about posters is that you try to communicate too much and end up obscuring the message." (P7)*	U
	**A8.6** Infographics need to be immediately readable and visually striking for patients passing through.	*"If you quickly walk past, it has to be visually strong." (P7)*	U
	**A8.7** Content should be adapted to individual patient needs.	*"I think it lacks more liquid nutrition options… something more realistic for my situation." (P15)*	U
	**A8.8** Identification with the symptom represented facilitates its expression.	*"If you walk by and think: am I the only one? Then it might become easier to put into words." (P7)*	U
	**A8.9** Infographics help reduce feelings of isolation related to symptoms.	*"If you walk by and think: am I the only one?" (P7)*	C
	**A8.10** Infographics encourage patients to discuss their symptoms with healthcare professionals.	*"You have to talk about it... You must tell the doctor and the nurses." (P6)*	U
	**A8.11** Infographics prepare patients for clinicians' questions and serve as "memory aids".	*"You'll be prepared to be asked the questions when you're with a nurse or a doctor." (P11)*	U
	**A8.12** Infographics prompt patients to request take-home support.	*"If it triggers some thoughts, it would be nice to have something to keep." (P7)*	U
**ART-9**	**A9.1** Some patients actively seek to understand and verify that the proposed treatment is the best option for them.	*"I want to know that what I'm doing is really the best opinion for me... Is there something else out there?" (P3, F, 40s)*	U
	**A9.2** Some patients describe a passive role and largely follow the oncologist's recommendations.	*"We pretty much followed his lead." (P7, M, 50s)* *"[Providers] decided to put me on this trial." (P10, F, 70s)*	U
	**A9.3** Some patients consult multiple specialists and travel long distances to access expertise or a recognised centre.	*"I took a trip to see Dr. and I also went to see another melanoma physician..." (P6, M, 70s)* *"I was going to [other medical center] … someone else to look at it and step back and say, 'Okay, this is what we are going to do'." (P1, M, 50s)*	U
	**A9.4** The risk of autoimmune side effects associated with treatment is perceived as disproportionate to expected benefits, leading some patients to refuse access to the innovation.	*"I looked at [this drug] really hard and I decided that it was not a drug that I would ever want to try. I do not like the autoimmune side effects that it has and the successes that they are having are just not worth, it's just not worth it." (P8, F, 30s)*	U
	**A9.5** Some patients rely on family members or close contact with medical expertise to help them understand therapeutic options.	*"My son-in-law is an aspiring medical researcher and my daughter is a nurse… they frequently provided information, advice, and research support." (P11, F, 50s)*	U
	**A9.6** Some patients actively seek clinical trials and travel specifically to assess their eligibility.	*"I came to [cancer center] with the hope of participating on a phase II clinical trial… 'roaring down' from my out-of-region home to determine if I was eligible. "(P4, F, 60s)*	U
	**A9.7** Clinical trials are sometimes viewed as a promising option, generating interest.	*"Yeah, [other clinic] is doing a clinical trial that I would be open to doing... they are getting some good results…" (P8, F, 30s)*	U
	**A9.8** Some patients maintain a highly optimistic view of their situation despite the perceived severity of the disease.	*"I am just a very lucky person. I have been so sick. I don't know how I have survived." (P2, M, 60s) ;* *"I know how bad my disease is. But… I feel good… I just know that it's going to have a good outcome." (P3, F, 40s)*	U
	**A9.9** Hope that the treatment is "better than nothing" motivates acceptance of experimental options.	*"We are doing this because we hope it will help... it's better than not getting anything." (P13, M, 40s)*	U
	**A9.10** A trusting relationship and good communication with the oncologist are perceived as very important.	*"I think he is a very good doctor. I think he really cares about me… and he's got a good sense of humor… I like him, I respect him." (P8, F, 30s) ; "[He] felt that [O] was always open to questions and that there was always plenty of time to ask and receive answers." (P6, M, 70s, fieldnote rapportant le vécu du patient)*	U
	**A9.11** Some patients describe shared decision-making with their oncologist.	*"Dr. always offered opinions and then we would decide together which way we would go." (P7, M, 50s)*	U
	**A9.12** Some patients describe decision-making with family members when relatives have a medical or advisory role.	*"We make all decisions together and in consultation with our son, who's a doctor also." (P12, M, 60s)*	U
	**A9.13** Some patients make decisions intuitively, relying on a feeling or inner conviction regarding the place or treatment.	*"I just felt like this was where I needed to go." (P3, F, 40s)*	U
	**A9.14** Some patients describe a rational decision-making strategy based on analysis of the benefits and risks of available options.	*"I looked at [this drug] really hard and I decided that it was not a drug that I would ever want to try… the successes that they are having are just not worth [the side effects]." (P8, F, 30s)*	U
	**A9.15** Some patients feel they have "no choice" regarding certain therapeutic options or trials.	*"No... Well, because I do not have any other alternative." (P10, F, 70s)*	U
	**A9.16** The desire to survive and prolong life is a central driver of treatment decisions, even when perceived as "frightening".	*"This ['scary' treatment] is just what you do because you want to live". (P8, F, 30s)* *"I definitely want to live for another year or 2..." (P2, M, 60s)*	U
	**A9.17** For some patients, quality of life outweighs certain therapeutic options perceived as too detrimental to cognitive functioning or identity.	*"It [brain radiation] makes you have no memory… I like to know who I am and who everybody around me is, and if I do not have that, what are you saving?" (P2, M, 60s)*	U

	**A9.18** Altruism, including the desire to help other patients or future generations, is an important motivation for trial participation.	*"Just wanting to know that someday what I did made a difference… whether it's my children or grandchildren or some girl in Haiti… that was definitely part of my thinking when I was considering a clinical trial." (P3, F, 40s)*	U
**ART-10**	**A10.1** Participation in the Remote Health Check (RHC) is motivated by a moral logic of giving, with patients wishing to contribute to the benefit of other patients even without expecting personal benefit.	*"I thought that this [RHC and research participation] was something I could do for you. To the benefit of others later." (P10)*<br>*"I had little expectations… something I could do for you. To the benefit of others later."*	U
	**A10.2** RHC questionnaires are sometimes difficult to understand.	*"It was very difficult to understand what you really want with these questions." (P7)*	U
	**A10.3** Patients lack clarity regarding available support or who to contact.	*"I got a phone number, but I don't know what's going on. If there's anyone who can help me at all." (P1)*	U
	**A10.4** The RHC does not allow precise expression of symptoms.	*"If I have pain, I don't get to elaborate on where the pain is when I tick the form." (P6)*	U
**ART-11**	**A11.1** Patients are motivated by the idea of rediscovering the pleasure of reading and reconnecting with a valued intellectual activity.	*"What motivated me to join in was the thought of enjoying the rewarding experience of reading books again…"*	U
	**A11.2** Fatigue, lack of motivation, and distractions limit physical activity, with audiobooks emerging as a more accessible alternative.	*"I find all sorts of distractions and excuses not to go out for a walk… I lack the motivation."*	U
	**A11.3** The group is perceived as a safe space to discuss literature rather than illness.	*"I would only really come along to the sessions because they involved discussing books and not talking about our illness."*	U
	**A11.4** Audiobooks carry strong emotional meaning and provide emotional support.	*"The audio books have been extremely meaningful to me."*	U
	**A11.5** Audio listening facilitates concentration by setting the pace of walking.	*"You can't focus on several things at once, you can only walk and listen."*	U
	**A11.6** Audiobooks offer a guided immersive experience that alters how patients perceive their journey.	*"You also get an interpretation of the book through the narrator, so you're guided through the book, which is quite interesting. It's an intense experience … Audio books make you see things in a different way."*	U
	**A11.7** Audiobooks help avoid the feeling of "wasting time" associated with traditional reading.	*"Sometimes when I sit down with a normal book I feel like I'm wasting time. But with audio books I don't get that feeling at all."*	U
	**A11.8** Audiobooks promote falling asleep and provide nighttime soothing, reducing bedtime-related anxiety.	*"I fall asleep more easily and I also feel more confident about going to bed…"*	U
	**A11.9** Literary exchanges within the group are experienced as rewarding and supportive.	*"Talking about what you've read together with others is very rewarding."*	U
	**A11.10** The programme creates a sense of commitment, framed as a positive obligation to read and listen.	*"It also made you feel obliged to read them too."*	U
	**A11.11** Reading enables patients to reaffirm an identity not reduced to cancer, relegating illness to a less central place.	*"The illness matters, but it's not the all-important thing."*	U
	**A11.12** Some literary content triggers discomfort when addressing darker themes.	*"You have to cling to hope. So it can be a bit difficult to cope with books involving numerous suicides…"*	U
	**A11.13** The intervention enables a rediscovery of the cultural pleasure of reading.	*"I have rediscovered the joy of literature again."*	U
	**A11.14** Books stimulate imagination and creativity.	*"A really good book is one that creates images that leave their mark."*	U
	**A11.15** The programme reinforces the notion of "taking care of oneself".	*"You're doing something good for yourself."*	U
	**A11.16** Activities outside clinical care (e.g., walking) provide relaxation, bodily awareness, and a sense of acting positively for oneself.	*"I could really feel myself relaxing my shoulders and breathing and I thought, 'You're doing something good for yourself'."*	U
	**A11.17** The combination of reading and walking benefits well-being and the perception of self-care.	*"It does something positive for your brain and for your legs too."*	U
	**A11.18** Outdoor activity provides relief and helps manage anxiety.	*"Getting outside has helped me – You shake off some of your worries when you walk outside."*	U
**ART-12**	**A12.1** Immersive virtual reality (iVR) is experienced as a discovery and sense of wonder, allowing exploration of unfamiliar places.	*"Certain places that I saw were beautiful, I didn't know them before" (ID31)*	U
	**A12.2** Visual immersion generates positive emotions related to colours, shapes, and the beauty of the virtual environment.	*"The colors are beautiful; the shapes are beautiful… Being able to admire the beautiful things that are there and around us."*	U
	**A12.3** iVR helps reduce anxiety during chemotherapy by providing complete distraction.	*"It helps calm you down during therapy and relax, it completely distracts you from all your anxieties."*	U
	**A12.4** Use of iVR alters time perception, making treatment sessions feel shorter.	*"The therapy went by much faster."*	U
	**A12.5** The device provides sensory immersion but requires adequate sound isolation to be effective.	*"It should allow you to isolate yourself completely without hearing external noises."*	C
	**A12.6** The VR headset is perceived as physically uncomfortable due to its weight.	*"The mask was very heavy, and I had to support it."*	U
	**A12.7** Use of iVR may be limited by lack of technical familiarity and difficulty handling the equipment.	*"I had problems precisely because of not knowing and not being familiar with the device."*	U
	**A12.8** iVR may cause visual fatigue or ocular discomfort.	*"There is also the strain on the eyesight…"*	U
**ART-13**	**A13.1** Access to information is perceived as clear, transparent, and well organised.	*"(the information) was definitely clear. They were pretty straightforward, and I received the information folders…" (P3)*	U
	**A13.2** Contradictory information between professionals generates uncertainty.	*"…there was one thing that was a bit unclear… whether there would be spinal anaesthesia. I got conflicting information…" (P8)*	U
	**A13.3** The decision to participate is based on the promise of rapid recovery, perceived as motivating.	*"My motivation… was that I was told that the recovery was very fast." (P6)*	U
	**A13.4** Sensations related to anaesthesia may cause bodily discomfort experienced negatively.	*"My whole body… the spinal anaesthesia was wearing off… I experienced that as an unpleasant feeling." (P3)*	U
	**A13.5** Walking independently to the operating theatre is experienced as empowering.	*"I liked the fact that I walked to the operating theatre… of course, I can walk…" (P1)*	U
	**A13.6** The active role of nursing staff (mobilisation, support) is experienced as helpful and reassuring.	*"A nurse came who intensively mobilized with me… she did that very well." (P2)*	U
	**A13.7** Early hospital discharge is not perceived as risky when patients trust the care team.	*"No, I don't think I got sick because I went home too early…" (P8)*	U
	**A13.8** Postoperative communication reassures patients and strengthens confidence in returning home.	*"The surgeon… 'how is it going?'… 'Good, then you can go home'. I also wanted to leave." (P2)*	U
	**A13.9** Access to healthcare professionals (call numbers, availability) enhances perceived safety.	*"If you have any complaints… always call. I was given two numbers…" (P9)*	U
	**A13.10** Patients feel confident when they believe teams would not allow discharge if there were any risk.	*"They don't let you go home anyway if something is not right, so I trusted that." (P4)*	U
	**A13.11** Telephone follow-up is experienced as a human continuation of care.	*"Yes, I liked it… aftercare is nice… you are not a number… you do matter." (P6)*	U
**ART-14**	**A14.1** Visual Education (VE) may generate emotional overload when provided too early in the care pathway.	*"It overwhelmed me."*	U
	**A14.2** Lack of practical information related to everyday life after treatment.	*"They said practical information 'on life after treatment', concerning daily activities, sexuality and sports, was missing."*	U
	**A14.3** VE is perceived as too simplified to support informed decision-making.	*"For me the EV was too simplistic… You really need to know your options before you can make a decision." (P6)*	U
	**A14.4** VE lacks sufficient depth to understand how the treatment works in the body.	*"It would have given me a better understanding of what really happened in my body."*	U
	**A14.5** VE supports decisional autonomy when it enables patients to inform themselves and independently weigh their options.	*"You have to take your own responsibility… you need to know your options before you can make a decision." (P6)*	U
	**A14.6** Some patients consider decision-making to remain personal and to require active information seeking beyond VE.	*"You need to look for answers yourself and weigh what really matters… and you decide in favour or against it." (P9)*	U
**ART-15**	**A15.1** Access to a digital platform fosters autonomy and emotional control.	*"It's not done after… it's a lifelong thing that we'll always re-engage in…" [S6F2]*<br>*"Having that available when people are ready to engage gives them access and gives them a sense of control…" [S6F2]*	U

	**A15.2** Connecting with other survivors validates their experiences and helps them feel understood.	*"Being able to connect to people that have gone through the same things as you… can validate your feelings…" [S4F5]*	U
	**A15.3** Social connections among survivors are experienced as therapeutic and as a source of community beyond illness.	*"Almost like a meet-up… talk about our stories… how we're moving forward…" [S4F3]*<br>*"Talk to people who have been through the same thing, but not talk about cancer at all." [S4F3]*	U
	**A15.4** A need for coherent and understandable follow-up with healthcare professionals to avoid post-treatment misunderstandings.	*"Did they hear that or did they just say, 'Worried about cancer but looks good'… I want to know that they had the same thing." [S1F4]*	U
	**A15.5** Access to information enables self-advocacy.	*"You're more involved if you have really easy access to all the information…" [S2F1]*<br>*"Including information on how to advocate for yourself would be important." [S3F3]*	U
	**A15.6** Survivors of childhood cancer seek tools to guide the transition to adult care and strengthen autonomy.	*"Something in aftercare… helping people advocate and engage in their care a little bit more…" [S3F4]*	U
	**A15.7** A digital summary helps communication and recall of details.	*"If that was in a digital form… that would be useful… customized to what treatment you had…" [S3F1]*	U
	**A15.8** Reviewing medical history promotes transparency and continuity.	*"…access to records, maybe notes of previous visits… something to refer to would be very convenient." [S1F1]*<br>*"Refer back to a visit… see how you've progressed…" [S1F1]*	U
	**A15.9** Appointment reminders, flexibility, and transport adaptations increase access and engagement.	*"I would love to have an appointment at a certain time… spending my whole day in clinic… those are the things that would make it easier for me to engage." [S3F1]*	U
	**A15.10** Access inequalities are substantial (rurality, transport, local availability); digital tools provide partial support.	*"Health equity is a dream, not a reality… care here is just not accessible…" [S4F5]*<br>*"Zoom and telecommunication… hopefully people are more open to doing these things virtually…" [S4F5] "If there was something to connect people who live remotely… that would help." [S3F5]*	U
	**A15.11** The ability to ask questions online to the care team facilitates access to follow-up.	*"A message feature would probably be efficient… instead of phone tag… on the app I could just send a message…" [S3F1]*	U
	**A15.12** Peer connections can be helpful, but "co-rumination" is perceived as a risk by some.	*"People who gravitate toward looking for support are people who aren't doing well… I don't want to be part of something that becomes a co-rumination." [S2F5]*	U
	**A15.13** Digital tools may compromise patient confidentiality when notifications are visible on shared family devices, inadvertently exposing sensitive information.	*"And I think it's not just me that you're kind of targeting with a platform like this — it's also especially for people who might be younger and still living at home. (…) With iPads and phones and emails and all these things now, if folks are still living at home, their parents may also be seeing these notifications." [S4F5]*	U
**ART-16**	**A16.1** Arrival at the innovative service is experienced as a turning point: after initially difficult access, reception is perceived as simple, clear, and reassuring.	*"Getting to the cancer service may have been challenging, once they arrived things were easier… 'straightforward after that.'" (Patient 2)*	U
	**A16.2** The human support provided by the care team is perceived as decisive, sometimes outweighing the perceived impact of medical treatment.	*"The support has been brilliant… helps me more than the chemo." (Patient 1)*	U
	**A16.3** Some staff members are perceived as going "beyond" their roles, strengthening trust and relational closeness.	*"The Nurse Unit Manager… stayed because my daughter and husband weren't around, and I think that is lovely." (Patient 5)*<br>*"Individual staff members… went 'above and beyond.'" (Patient 3)*	U
	**A16.4** Clear, personalised, and accessible communication facilitates navigation within the system and enhances understanding of the care pathway.	*"They explained everything… I knew where to go and how to go about it." (Patient 4)*<br>*"The information… has been phenomenal… my family too received the information." (Patient 1)*	U
	**A16.5** The pathway prior to accessing the innovative service is described as confusing and stressful, marked by delays, diagnostic errors, and poor communication.	*"It was confusing, stressful, and marked by medical delays, misdiagnosis and poor communication."*<br>*"I had three different doctors… it had taken well over a month to get anything back." (Patient 2)*<br>*"They told me I had glandular fever… I just want to die." (Patient 5)*	U
	**A16.6** Family support is perceived as essential for emotional support and continuation of care.	*"I don't know if I would still be here if I was on my own." (Patient 4)*<br>*"It makes everything just so much easier." (Patient 1)*<br>*"Another pair of ears that understands." (Patient 2)*	U
**ART-17**	**A17.1** Oral anticancer treatment (OAT) alters body image and serves as a daily reminder of the illness.	*« On se reconnaît plus dans la glace… ça nous rappelle tous les jours qu'on est malade. »*<br>*« J'en ai ras le bol du corps, envahi par tous ces produits chimiques. »*	C
	**A17.2** The disease symbolises chronicity and ongoing uncertainty.	*« C'est une épée de Damoclès qui plane sur la tête. »*<br>*« C'est une maladie chronique, la preuve : j'ai arrêté 15 jours et c'était reparti. »*	C
	**A17.3** Patients compare OAT with injectable treatments in terms of side effects.	*« C'est des pochettes de produit qu'on vous injecte… tandis que mes cachets empêchent mon corps de fabriquer une protéine. »*<br>*« Le cachet, c'est moins difficile à supporter. »*	C
	**A17.4** Oral administration involves a mental burden related to daily intake.	*« Des fois, le week-end le matin, j'éteins mon réveil et… je me retourne quoi. »*<br>*« C'est la charge mentale du traitement oral. »*	C
	**A17.5** OAT modifies the relationship with the hospital and follow-up care: relief at avoiding hospital visits but a sense of loneliness.	*« Ne pas aller à l'hôpital, c'est un soulagement. »*<br>*« Sans le contact avec les infirmiers, je me sens plus seule. »*<br>*« J'ai perdu le bouton rouge. »*	C
	**A17.6** OAT requires a sometimes constrained form of self-management, with patients managing on their own and assuming responsibility for treatment.	*« La nécessité de se prendre en mains. »*<br>*« Se gérer tout seul. »*	C
**ART-18**	**A18.1** Access to the trial is experienced as a therapeutic opportunity not to be missed, under time pressure.	*« Si vous décidez dans quatre mois on ne peut pas garantir qu'il reste de la place. »*<br>*« Faut peut-être pas que je laisse passer ma chance. »*	C
	**A18.2** Being able to reconsider one's decision is reassuring and facilitates choice.	*« On arrêtera l'étude à tout moment si… »*	C
	**A18.3** Trust in the oncologist is decisive: the trial is accepted because it is proposed by them.	*« J'ai eu son portable tout de suite… pouvoir appeler à 3 h du matin. »*<br>*« Si c'est elle qui me le propose, c'est que c'est bien pour moi. »*<br>*« On me connaît, on m'appelle par mon prénom. »*	C
	**A18.4** Consent is sometimes signed very quickly, occasionally without full reading, in a context of emotional urgency.	*« J'ai signé tout de suite, sans vraiment lire. »*<br>*« On m'a demandé de signer le jour même. »*	C
	**A18.5** Some patients do not fully perceive the implications of signing.	*« Je crois qu'ils n'ont pas très bien compris l'importance de la signature. »*	C
	**A18.6** The trial is experienced as the absence of therapeutic alternatives, with a feeling of having "no choice".	*« On m'a dit : y a pas d'autre solution. Donc j'ai pas le choix. »*<br>*« Dans la maladie, on a peu de choix. »*	C
	**A18.7** The clinical trial is invested with hope, enabling patients to "hold on for others", particularly for family members.	*« Je fais ça pour mes filles. »*	C
	**A18.8** Moral conflicts with relatives (partner, physician friends) arise around participation.	*« Lui, il était contre. »*<br>*« Mes amis médecins m'ont dit de ne pas le faire. »*	C
	**A18.9** The trial reinforces a sense of being recognised, supported, and surrounded within the research unit.	*« Je me sens comme chez moi. »*<br>*« Le médecin m'a dit : appelez-moi n'importe quand. »*	C
	**A18.10** The trial is interpreted within a moral logic of giving and reciprocity, contributing to supporting research.	*« C'est comme donner son sang. »*<br>*« On a bénéficié de médicaments testés avant nous, il faut aider la recherche à avancer. »*	C
	**A18.11** Trial-related side effects are accepted as the lesser evil, despite uncertainty.	*« Si y a des petits effets secondaires, c'est un moindre mal. »*	C
	**A18.12** The trial repairs a previously experienced sense of abandonment within the healthcare system.	*« C'est fini, on me laisse tomber. »*<br>*« Je me sentais abandonnée. »*	C
	**A18.13** Emotional attachment to the care team may be ambivalent and experienced as constrained.	*« Je m'attache, mais faut pas, c'est plus fort que moi. »*	C
	**A18.14** After multiple lines of treatment, some patients feel ready to enter a trial.	*« J'avais plus peur, j'avais déjà maturé tout ça. »*	C

	**A18.15** The clinical trial is perceived as unexpected and generates immediate acceptance.	*« C'était inespéré ; j'étais partante tout de suite. »*	C
**ART-19**	**A19.1** Access to targeted therapies is perceived as a temporary chance to prolong life, without illusion of cure.	*"I don't deceive myself that it's going to cure me… I don't know if… it's just holding it at bay." (Karen, 71; Francis, 69)*<br>*"At least it gives me a few more years." (Olivia, 67)*	U
	**A19.2** Patients experience a suspended temporality, oscillating between hope and resignation.	*"So future is more like a present. I just live through it… I enjoy it." (Binh, 44)*<br>*"I live my life day by day… no sense of hope." (Luca, 49)*<br>*"It's probably given me a bit more hope for time." (Melissa, 55)*	U
	**A19.3** Treatment is experienced as ambiguous, between life prolongation and absence of cure.	*"I start to feel well… but I can't be sure… now I have hope." (Antonia, 61)*<br>*"My doctors aren't completely sure… but it's keeping me well." (Rosie, 56)*	U
	**A19.4** Uncertainty related to scans ("scanxiety") affects everyday life.	*"The scan is going to screw me right up… results the next day, you're fine." (Luca, 49)*<br>*"The report is late… you just hang in there." (Claire, 64)*<br>*"If I was coming in… and they said, 'This has grown'… I don't want to anymore scans." (Nadine, 54)*	U
	**A19.5** Targeted therapies are perceived as effective but unstable, with a limited duration over time.	*"Those drugs only work for a certain time… then I got a progression." (Binh, 44)*<br>*"I think the cancer has finally caught up with me." (Duy, 59)*	U
	**A19.6** The body becomes dependent on the medication, sometimes experienced as being "enslaved to the treatment".	*"You think you become a slave of the medicine." (Ranjeet, 65)*	U
	**A19.7** Side effects affect body image.	*"Feet peeling like a snakeskin… maybe forever." (Vincent, 79)*<br>*"You're not sure whether it's a side effect or not…" (Rosie, 56)*	U
	**A19.8** Side effects come to be perceived as a "price to pay".	*"If you can work around these side effects… you can cope a lot better." (Hannah, 64)*	U
	**A19.9** Precision medicine is accompanied by a sense of existential exhaustion.	*"[I feel I'm] just surviving… you don't have any motivation… breathing without any soul." (Ranjeet, 65)*	U
	**A19.10** Some patients feel isolated due to the rarity of their cancer.	*"I live my life day by day… as a minority in the cancer world." (Luca, 49)*	U
	**A19.11** Targeted therapies modify perceptions of the body, being experienced as a biological device that "contains" and controls the cancer.	*"It won't allow the cancer to go places… it's managed it." (Claire, 64)*<br>*"The cancer cell, it's covered, and my body works and kills this cell." (Antonia, 61)*	C
**ART-20**	**A20.1** RT Prepare (Radiotherapy Prepare Program) is perceived as a privileged space for listening, allowing patients to express concerns and feel reassured before radiotherapy.	*"Just to sit down with somebody and voice a couple of concerns was most helpful."*<br>*"It allayed fears… I felt a bit more prepared."*	U
	**A20.2** Understanding the treatment facilitates emotional and cognitive preparation.	*"Better to know what it does and what it looks like before you go in there."*	U
	**A20.3** Repetition of explanations strengthens confidence.	*"Even if three or four different people did it… that gave me confidence."*	U
	**A20.4** Patients value dedicated and sufficient time, enabling them to process information and ask questions.	*"They dedicated that time… it wasn't rushed."*	U
	**A20.5** There is a need for written materials to retain practical advice for home care.	*"A little pamphlet that gives you some idea of skin care would help when you get home."*	U
	**A20.6** A calm, private, and appropriate environment (e.g., a room with a window) enhances feelings of reassurance.	*"The second meeting had a window… I felt more reassured."*	C

Web Appendix Table 5: Critical Appraisal Skills Programme (CASP)quality appraisal of included studies

Étude	CASP1	CASP2	CASP3	CASP4	CASP5	CASP6	CASP7	CASP8	CASP9	CASP10	Overall appraisal	Comments (Strengths /Limitations / Unknowns)
**Myren et al., 2020**	Y	Y	Y	Y	Y	Y	Y	Y	Y	Y	High	**Strengths:** Structured qualitative methodology; double coding; explicit analytical framework. **Limitations:** Sample focused on gynaecological oncology; limited reflexivity; saturation and transferability not reported.
**Cusimano et al., 2020**	Y	Y	Y	Y	Y	Y	Y	Y	Y	Y	High	**Strengths:** Rigorous qualitative design; strong contextualisation. **Limitations:** Single-centre study; predominantly descriptive interpretive level; interview duration and saturation not reported.
**Matthew et al., 2025**	Y	Y	Y	Y	Y	CT	Y	Y	Y	Y	High	**Strengths:** Integration of patients and healthcare professionals; rich data; saturation achieved. **Limitations:** Reflexivity not addressed; short interviews; influence of institutional context and participant diversity not clearly described.
**Ranes et al., 2022**	Y	Y	Y	Y	Y	CT	Y	Y	Y	Y	High	**Strengths:** Qualitative design with triangulation of expertise and transparent methodology. **Limitations:** Small sample size; absence of participant validation; verbatim selection process and analytical tools not described.
**Kristensen et al., 2024**	Y	Y	Y	Y	Y	N	Y	Y	Y	CT	High	**Strengths:** Coherent and well-described methodology. **Limitations:** Small sample size; no mention of saturation; limited scope of perspectives and generalisability.
**Ala-Leppilampi et al., 2020**	Y	Y	Y	Y	Y	N	Y	Y	Y	Y	High	**Strengths:** Clear objectives and original contribution on the side effects of immune checkpoint inhibitors (ICIs). **Limitations:** Limited reflexivity and English-speaking single-centre setting restricting transferability; participant validation and sociocultural context not reported.
**Wang et al., 2022**	Y	CT	Y	Y	Y	N	Y	CT	Y	Y	Moderate	**Strengths:** Structured mixed-methods approach with a rigorous process. **Limitations:** Limited reflexivity and single-centre study; absence of participant validation and lack of detail on inter-analyst coding.
**Piil et al., 2023**	Y	Y	Y	Y	Y	N	Y	Y	Y	Y	Moderate	**Strengths:** Clear objectives, coherent design, and multi-analyst analysis. **Limitations:** Reflexivity not addressed and small sample with predominantly descriptive findings; participant validation and transparency regarding quote selection not reported.
**Garrett et al., 2018**	Y	Y	Y	Y	Y	N	–	Y	Y	Y	High	**Strengths:** Methodologically robust ethnography with double coding and in-depth analysis. **Limitations:** Reflexivity not addressed and single-centre, limited diversity sample; saturation, participant validation, and transferability not reported.
**Oelschlägel et al., 2024**	Y	Y	Y	Y	Y	CT	Y	Y	Y	Y	High	**Strengths:** Robust methodology and clinical usefulness. **Limitations:** Reflexivity not addressed and saturation not explicitly reported; uncertain transferability and influence of the COVID context not evaluated.
**Hammer et al., 2017**	Y	Y	Y	CT	Y	CT	Y	Y	Y	Y	High	**Strengths:** Solid design and multi-method data collection. **Limitations:** Limited discussion and saturation not reported; generalisability, potential recruitment biases, and social desirability bias not clearly addressed.
**Burrai et al., 2025**	Y	Y	CT	Y	Y	CT	Y	Y	Y	Y	High	**Strengths:** Rich data and coherent application of a theoretical framework. **Limitations:** Authors' reflexivity not discussed; participant validation of interpretations not reported.
**Sier et al., 2024**	Y	Y	Y	Y	Y	Y	Y	Y	Y	Y	High	**Strengths:** Coherent mixed-methods design with saturation achieved and independent double coding. **Limitations:** Single-centre study and small sample without participant validation; effects of the institutional context and postoperative timing not discussed.
**Wolters-Zwolle et al., 2022**	Y	Y	Y	Y	Y	Y	Y	Y	Y	Y	High	**Strengths:** Robust qualitative methodology with triangulation and demonstrated saturation. **Limitations:** Limited sample in a single setting and absence of participant validation; long-term impact and

												international/digital transferability not explored.
**Hou et al., 2024**	Y	Y	Y	Y	Y	Y	Y	Y	Y	Y	High	**Strengths:** Participatory approach with multi-informant triangulation and cross-validation. **Limitations:** Predominantly female sample and online data collection limiting depth; real-world impact and transferability beyond English-speaking Canadian populations not evaluated.
**Taylor et al., 2021**	Y	Y	Y	Y	Y	Y	Y	Y	Y	Y	High	**Strengths:** Culturally grounded ethical approach with community validation. **Limitations:** Sample limited to two high-performing services and partial saturation on the patient side; absence of non-adherent perspectives and generalisability not evaluated.
**Marmorat, 2018**	Y	Y	Y	CT	CT	N	N	CT	Y	CT	Moderate	**Strengths:** Clear conceptualisation and coherent thematic analysis. **Limitations:** Reflexivity, triangulation, and saturation not reported; limited recruitment and transferability beyond the ONCORAL context not specified.
**Derbez, 2015**	Y	Y	Y	CT	Y	Y	CT	Y	Y	Y	High	**Strengths:** Contextualised ethnography with strong reflexivity and innovative analysis. **Limitations:** No mention of ethics committee approval and loosely formalised recruitment; number of interviews, consent procedures, and access to raw data not reported.
**Flore, 2023**	Y	Y	Y	CT	CT	N	Y	Y	Y	Y	Moderate	**Strengths:** Coherent methodology with iterative analysis. **Limitations:** Reflexivity not addressed and limited justification of the design and interview guide; methodological adaptations and researcher influence not clearly described.
Halkett et al., 2020	Y	Y	Y	Y	CT	N	Y	CT	Y	Y	Moderate	**Strengths:** Rigorous recruitment and structured thematic analysis. **Limitations:** Limited reflexivity and partial transparency of the analytical process; refusals/non-participation and researcher or site influence not documented.

Y = Yes, N = No, CT = Can't tell (insufficient or unreported information)

Overall appraisal:
High indicates strong methodological coherence and transparency
Moderate indicates some limitations that may affect confidence in the findings
Low indicates major methodological limitations likely to compromise credibility

CASP1: Was there a clear statement of the aims of the research?
CASP2 : Is a qualitative methodology appropriate?
CASP3 : Was the research design appropriate to address the aims of the research?
CASP4 :Was the recruitment strategy appropriate to the aims of the research?
CASP5 : Was the data collected in a way that addressed the research issue?
CASP6 : Has the relationship between researcher and participants been adequately considered?
CASP7 : Have ethical issues been taken into consideration?
CASP8 : Was the data analysis sufficiently rigorous?
CASP9 : Is there a clear statement of findings?
CASP10 : How valuable is the research?

Web Appendix Table 6: Summary of Findings and Confidence in the Qualitative research (ConQual)assessments

Synthesized Finding	Type of studies	Dependability	Credibility	Final ConQual
SF 1: Understanding of innovations was described as facilitated when information was shared over time, using accessible language and consultable formats. These formats allowed patients to revisit information according to their availability, which was shaped by fatigue, emotional burden, or their clinical condition at a given moment.	Qualitative	Downgrade −1 (limited reflexivity and insufficient reporting of saturation across several contributing studies).	Downgrade -1 (Mix U/C)	Low
SF 2: Access to innovation was described as depending on the quality of care relationships, the continuity of interactions, and patients' ability to orient themselves within a care pathway that was sometimes perceived as uncertain.	Qualitative	No downgrade	Downgrade -1 (Mix U/C)	Moderate
SF 3: When innovation relied on self-management, patients experienced it as a source of control over symptoms and of personal validation, but also as an additional responsibility. Depending on the availability of support and individual resources, it could generate feelings of isolation, cognitive burden, or anxiety.	Qualitative	Downgrade −1 (recurrent methodological limitations related to saturation, reflexivity, and participant validation).	Downgrade -1 (Mix U/C)	Low
SF4: Patients sought spaces of respite within the care pathway, allowing them to reconnect with social, cultural, or sensory dimensions that were not centred on the disease.	Qualitative	No downgrade	Downgrade -1 (Mix U/C)	Moderate

Dependability was assessed using five CASP items aligned with JBI guidance (CASP 3, 4, 5, 8, and 9), covering study design, recruitment strategy, data collection, analytical rigour, and clarity of findings. Downgrading was applied when recurrent uncertainties across contributing studies were identified in one or more of these domains.
Credibility was downgraded when synthesised findings were supported by a combination of unequivocal (U) and credible (C) findings. No downgrading was applied when synthesised findings were supported exclusively by unequivocal findings.

Web Appendix Figure 1: Visual representation of synthesised findings

Findings	Category	Synthetized findings
**A1.1 (U)** Participation enables patients to understand teamwork, improving trust.<br>**A1.2 (U)** Participation is perceived as helping patients better understand side effects and reduce emotional burden.<br>**A1.3 (C)** Adapting medical language is necessary to ensure understanding.<br>**A2.3 (C)** The innovation improves understanding of end-of-life care.<br>**A3.1 (U)** Sexual health is not initiated by healthcare professionals; patients must seek information themselves.<br>**A3.3 (U)** Patients wish Sexual Health Care (SHC) to be introduced at an appropriate time (after initial treatment).<br>**A7.14 (U)** Nursing advice improves tolerance of oral nutritional supplements (ONS).<br>**A7.18 (U)** Patients consider individual consultations the best way to receive personalised information, but perceive them as inaccessible due to limited human resources.<br>**A8.4 (U)** Fatigue, limited concentration, and visual size influence the ability to understand infographics.<br>**A10.2 (U)** Remote Health Check (RHC) questionnaires are sometimes difficult to understand.<br>**A10.4 (U)** The RHC does not allow precise expression of symptoms.<br>**A13.1 (U)** Access to information is perceived as clear, transparent, and well organised.<br>**A13.2 (U)** Contradictory information between professionals generates uncertainty.<br>**A14.2 (U)** Lack of practical information related to everyday life after treatment.<br>**A14.3 (U)** Visual Education (VE) is perceived as too simplified to support informed decision-making.<br>**A14.4 (U)** VE lacks sufficient depth to understand how the treatment works in the body.<br>**A20.2 (U)** Understanding the treatment facilitates emotional and cognitive preparation.<br>**A20.3 (U)** Repetition of explanations strengthens confidence.<br>**A20.5 (U)** There is a need for written materials to retain practical advice for home care.	1. Understanding information related to innovative care	1: Understanding of innovations was described as facilitated when information was shared over time, using accessible language and consultable formats. These formats allowed patients to revisit information according to their availability, which was shaped by fatigue, emotional burden, or their clinical condition at a given moment.
**A2.6 (U)** A persistent fear of disease progression and death remains despite support.<br>**A2.7 (U)** Palliative care is associated with a poor prognosis.<br>**A5.1 (U)** The diagnosis is experienced as a major shock.<br>**A5.3 (U)** Emotional burden hinders understanding of information.<br>**A5.4 (U)** Patients experience information overload at the end of consultations.<br>**A5.5 (U)** Radiotherapy is presented as a difficult treatment, reinforcing emotional burden.<br>**A5.10 (U)** Waiting before treatment is difficult to accept.<br>**A6.5 (U)** Hypervigilance regarding side effects.<br>**A6.6 (U)** Anxiety related to new treatments.<br>**A12.3 (U)** Immersive virtual reality (iVR) helps reduce anxiety during chemotherapy by providing complete distraction.<br>**A14.1 (U)** Visual Education (VE) may generate emotional overload when delivered too early in the care pathway.<br>**A19.2 (U)** Patients experience a suspended temporality, oscillating between hope and resignation.<br>**A19.3 (U)** Treatment is experienced as ambiguous, between life prolongation and absence of cure.<br>**A19.4 (U)** Uncertainty related to scans ("scanxiety") affects everyday life.<br>**A19.9 (U)** Precision medicine is accompanied by a sense of existential exhaustion.<br>**A20.1 (U)** RT Prepare (Radiotherapy Prepare Program) is perceived as a privileged space for listening, allowing patients to express concerns and feel reassured before radiotherapy.	2. Uncertainty and concerns related to innovative treatments	
**A6.1 (U)** Profound and indescribable fatigue.<br>**A6.2 (U)** Perceived physical decline and loss of former identity.<br>**A6.3 (U)** Intense itching.<br>**A6.4 (U)** Generalised pain and loss of mobility.<br>**A6.8 (U)** Difficulty attributing symptoms.<br>**A6.9 (U)** Side effects perceived as signs of treatment effectiveness.<br>**A6.10 (U)** Hope persists despite the severity of side effects.<br>**A7.27 (U)** Discontinuation of oral nutritional supplements occurs when patients do not perceive concrete benefits (e.g., weight maintenance).<br>**A12.6 (U)** The VR headset is perceived as physically uncomfortable due to its weight.<br>**A12.8 (U)** iVR may cause visual fatigue or ocular discomfort.<br>**A13.4 (U)** Sensations related to anaesthesia may cause bodily discomfort experienced negatively.<br>**A17.1 (C)** Oral treatment alters body image and serves as a daily reminder of the illness.<br>**A19.7 (U)** Side effects affect body image.<br>**A19.8 (U)** Side effects come to be perceived as a "price to pay".	7. Bodily manifestations related to illness and treatments	
**A3.4 (U)** Visual supports facilitate the introduction of Sexual Health Care (SHC).<br>**A7.14 (U)** Nursing advice improves tolerance of oral nutritional supplements.<br>**A7.15 (U)** Educational presentations are highly requested.<br>**A7.16 (U)** The WeChat nutrition group is perceived as helpful in avoiding errors and better understanding recommendations.<br>**A7.17 (U)** Patients request simple guides (PDFs, e-books).<br>**A7.23 (U)** Patients want educational videos delivered via WeChat.<br>**A7.25 (U)** Videos reduce the need for repeated follow-up.<br>**A8.1 (U)** Infographic colours elicit emotions and personal associations that influence interpretation.<br>**A8.2 (U)** Some images are difficult to interpret (shapes, symbols, colours), hindering understanding.<br>**A8.3 (U)** Some images evoke hope and a more positive future.<br>**A8.4 (U)** Fatigue, limited concentration, and visual size influence the ability to understand infographics.<br>**A8.5 (U)** Excessive text or information reduces message impact and readability.<br>**A8.6 (U)** Infographics need to be immediately readable and visually striking for patients passing through.<br>**A8.7 (U)** Content should be adapted to individual patient needs.<br>**A8.8 (U)** Identification with the symptom represented facilitates its expression.<br>**A8.9 (C)** Infographics help reduce feelings of isolation related to symptoms.<br>**A8.10 (U)** Infographics encourage patients to discuss their symptoms with healthcare professionals.<br>**A8.11 (U)** Infographics prepare patients for clinicians' questions and serve as "memory aids".<br>**A8.12 (U)** Infographics prompt patients to request a take-home support.<br>**A15.1 (U)** Access to a digital platform fosters autonomy and emotional control.<br>**A15.5 (U)** Access to information enables self-advocacy.<br>**A15.7 (U)** A digital summary supports communication and recall of details.<br>**A15.8 (U)** Reviewing medical history promotes transparency and continuity.<br>**A20.5 (U)** There is a need for written materials to retain practical advice for home care.	11. Supportive tools that help navigate the care pathway	

Findings	Category	Synthetized findings
**A2.2 (U)** Access to the programme provides emotional security and support, alleviating fear and loneliness.<br>**A2.5 (U)** Relational continuity transforms fear into trust.<br>**A2.8 (C)** The SMS programme provides reassuring and accessible support, strengthening the sense of security.<br>**A3.2 (U)** Perceived lack of time during consultations hinders discussion of sexual health.<br>**A4.2 (U)** The group is experienced as a supportive and motivating community.<br>**A5.7 (U)** Home and family support are essential.<br>**A6.12 (U)** Support from healthcare professionals is essential.<br>**A6.13 (U)** Family and social support are decisive.	3. Being supported and recognised by healthcare professionals and relatives	2: Access to innovation was described as depending on the quality of care relationships, the continuity of interactions, and

**A7.19 (U)** Patients seek regular follow-up (monthly or bimonthly) as it reinforces feelings of support.<br>**A7.20 (U)** Patients consider very frequent follow-up, particularly early in the pathway, unnecessary and not beneficial.<br>**A7.22 (U)** Face-to-face encounters are perceived as more reassuring and allow professionals to consider the person holistically.<br>**A7.24 (U)** Patients describe an ideal follow-up duration of 1–6 months, with several regular contacts fostering continuity of care.<br>**A7.26 (U)** Family members facilitate acceptance of oral nutritional supplements by repeating and re-explaining information provided by healthcare professionals.<br>**A7.28 (U)** Family support encourages continuation of oral nutritional supplements.<br>**A7.29 (U)** Dietitian reminders promote long-term adherence.<br>**A13.6 (U)** The active role of nursing staff (mobilisation, support) is experienced as helpful and reassuring.<br>**A13.7 (U)** Early hospital discharge is not perceived as risky when patients trust the care team.<br>**A13.8 (U)** Postoperative communication reassures patients and strengthens confidence in returning home.<br>**A13.9 (U)** Access to healthcare professionals (call numbers, availability) enhances perceived safety.<br>**A13.10 (U)** Patients feel confident when they believe teams would not allow discharge if there were any risk.<br>**A13.11 (U)** Telephone follow-up is experienced as a human continuation of care.<br>**A15.4 (U)** There is a need for coherent and understandable follow-up with healthcare professionals to avoid post-treatment misunderstandings.<br>**A15.11 (U)** The ability to ask questions online to the care team facilitates access to follow-up.<br>**A16.2 (U)** Human support provided by the care team is perceived as decisive, sometimes outweighing the perceived impact of medical treatment.<br>**A16.3 (U)** Some staff members are perceived as going "beyond" their roles, strengthening trust and relational closeness.<br>**A16.6 (U)** Family support is perceived as essential for emotional support and continuation of care.<br>**A18.3 (C)** Trust in the oncologist is decisive: the trial is accepted because it is proposed by them.<br>**A18.9 (C)** The trial reinforces a sense of being recognised, supported, and surrounded within the research unit.<br>**A18.12 (C)** The trial repairs a previously experienced sense of abandonment within the healthcare system.<br>**A18.13 (C)** Emotional attachment to the care team may be ambivalent and experienced as constrained.<br>**A18.15 (C)** The clinical trial is perceived as unexpected and generates immediate acceptance.<br>**A20.1 (U)** RT Prepare (Radiotherapy Prepare Program) is perceived as a privileged space for listening, allowing patients to express concerns and feel reassured before radiotherapy.<br>**A20.4 (U)** Patients value dedicated and sufficient time, enabling them to process information and ask questions.<br>**A20.6 (C)** A calm, private, and appropriate environment (e.g., a room with a window) enhances feelings of reassurance.		patients' ability to orient themselves within a care pathway that was sometimes perceived as uncertain.
**A5.6 (U)** Proximity to the centre facilitates access.<br>**A7.21 (U)** Limitations in digital use among some patients lead them to favour direct channels (e.g., telephone), perceived as more accessible than technological innovations.<br>**A10.3 (U)** Patients lack clarity regarding available support or who to contact.<br>**A15.9 (U)** Appointment reminders, flexibility, and transport adaptations facilitate access and engagement.<br>**A15.10 (U)** Access inequalities are substantial (rurality, transport, local availability); digital tools provide partial support.<br>**A16.1 (U)** Arrival at the innovative service is experienced as a turning point: after initially difficult access, reception is perceived as simple, clear, and reassuring.<br>**A16.4 (U)** Clear, personalised, and accessible communication facilitates navigation within the system and enhances understanding of the care pathway.	5. Accessing services and navigating the organisation of care	
**A5.2 (U)** Immediate priority is survival rather than participation in the RCT.<br>**A5.8 (U)** Lack of evidence on proton therapy generates uncertainty.<br>**A5.9 (U)** Randomisation is experienced as a loss of control.<br>**A7.12 (U)** A physician's recommendation leads to strong adherence.<br>**A9.1 (U)** Some patients actively seek to understand and verify that the proposed treatment is the best option for them.<br>**A9.2 (U)** Some patients describe a passive role and largely follow the oncologist's recommendations.<br>**A9.3 (U)** Some patients consult multiple specialists and travel long distances to access expertise or a recognised centre.<br>**A9.4 (U)** The risk of autoimmune side effects is perceived as disproportionate to expected benefits, leading some patients to refuse access to the innovation.<br>**A9.5 (U)** Some patients rely on family members or close contacts with medical expertise to help them understand therapeutic options.<br>**A9.6 (U)** Some patients actively seek clinical trials and travel specifically to assess eligibility.<br>**A9.7 (U)** Clinical trials are sometimes viewed as a promising option, generating interest.<br>**A9.8 (U)** Some patients maintain a highly optimistic view of their situation despite the perceived severity of the disease.<br>**A9.9 (U)** Hope that treatment is "better than nothing" motivates acceptance of experimental options.<br>**A9.10 (U)** A trusting relationship and good communication with the oncologist are perceived as very important.<br>**A9.11 (U)** Some patients describe shared decision-making with their oncologist.<br>**A9.12 (U)** Some patients describe decision-making with family members when relatives have a medical or advisory role.<br>**A9.13 (U)** Some patients make decisions intuitively, relying on a feeling or inner conviction regarding the place or treatment.<br>**A9.14 (U)** Some patients describe a rational decision-making strategy based on analysis of benefits and risks.<br>**A9.15 (U)** Some patients feel they have "no choice" regarding certain therapeutic options or trials.<br>**A9.16 (U)** The desire to survive and prolong life is a central driver of treatment decisions, even when perceived as frightening.<br>**A9.17 (U)** For some patients, quality of life outweighs therapeutic options perceived as too detrimental to cognitive functioning or identity.<br>**A9.18 (U)** Altruism—the desire to help other patients or future generations—is an important motivation for trial participation.<br>**A10.1 (C)** Participation in the Remote Health Check (RHC) is motivated by a moral logic of giving, with patients wishing to benefit others even without expecting personal benefit.<br>**A13.3 (U)** The decision to participate is based on the promise of rapid recovery, perceived as motivating.<br>**A14.5 (U)** Visual Education (VE) supports decisional autonomy when it enables patients to inform themselves and independently weigh options.<br>**A14.6 (U)** Some patients consider decision-making to remain personal and to require active information seeking beyond VE.<br>**A17.3 (C)** Patients compare oral anticancer treatments with injectable therapies in terms of side effects.<br>**A18.1 (C)** Access to the trial is experienced as a therapeutic opportunity not to be missed, under time pressure.<br>**A18.2 (C)** Being able to reconsider one's decision is reassuring and facilitates choice.<br>**A18.4 (C)** Consent is sometimes signed very quickly, occasionally without full reading, in a context of emotional urgency.<br>**A18.5 (C)** Some patients do not fully perceive the implications of signing consent.<br>**A18.6 (C)** The trial is experienced as the absence of therapeutic alternatives, with a feeling of having "no choice".<br>**A18.7 (C)** The trial is invested with hope, enabling patients to "hold on for others", particularly family members.<br>**A18.8 (C)** Moral conflicts with relatives (partners, physician friends) arise around participation.<br>**A18.10 (C)** The trial is interpreted within a moral logic of giving and reciprocity in support of research.<br>**A18.11 (C)** Trial-related side effects are accepted as the lesser evil, despite uncertainty.<br>**A18.14 (C)** After multiple lines of treatment, some patients feel ready to enter a trial.<br>**A19.1 (U)** Access to targeted therapies is perceived as a temporary chance to prolong life, without illusion of cure.<br>**A19.5 (U)** Targeted therapies are perceived as effective but unstable, with a limited duration over time.	6. Making decisions between therapeutic options	
**A6.7 (C)** A sense of abandonment.<br>**A13.10 (U)** Patients feel confident when they believe that care teams would not allow discharge if there were any risk.<br>**A16.5 (U)** The pathway prior to accessing the innovative service is described as confusing and stressful, marked by delays, diagnostic errors, and poor communication.<br>**A18.9 (C)** The trial reinforces a sense of being recognised, supported, and surrounded within the research unit.<br>**A18.12 (C)** The trial repairs a previously experienced sense of abandonment within the healthcare system.<br>**A20.1 (U)** RT Prepare (Radiotherapy Prepare Program) is perceived as a privileged space for listening, allowing patients to express concerns and feel reassured before radiotherapy.<br>**A20.6 (C)** A calm, private, and appropriate environment (e.g., a room with a window) enhances feelings of reassurance.	13. Trust and disruption in the relationship with the healthcare system	

Findings	Category	Synthetized findings
**A2.6 (U)** A persistent fear of disease progression and death remains despite support.<br>**A2.7 (U)** Palliative care is associated with a poor prognosis.<br>**A5.1 (U)** The diagnosis is experienced as a major shock.<br>**A5.3 (U)** Emotional burden hinders understanding of information.<br>**A5.4 (U)** Patients experience information overload at the end of consultations.<br>**A5.5 (U)** Radiotherapy is presented as a difficult treatment, reinforcing emotional burden.<br>**A5.10 (U)** Waiting before treatment is difficult to accept.<br>**A6.5 (U)** Hypervigilance regarding side effects.<br>**A6.6 (U)** Anxiety related to new treatments.<br>**A12.3 (U)** Immersive virtual reality (iVR) helps reduce anxiety during chemotherapy by providing complete distraction.<br>**A14.1 (U)** Visual Education (VE) may generate emotional overload when delivered too early in the care pathway.<br>**A19.2 (U)** Patients experience a suspended temporality, oscillating between hope and resignation.<br>**A19.3 (U)** Treatment is experienced as ambiguous, between life prolongation and absence of cure.<br>**A19.4 (U)** Uncertainty related to scans ("scanxiety") affects everyday life.<br>**A19.9 (U)** Precision medicine is accompanied by a sense of existential exhaustion.<br>**A20.1 (U)** RT Prepare (Radiotherapy Prepare Program) is perceived as a privileged space for listening, allowing patients to express concerns and feel reassured before radiotherapy.	2. Uncertainty and concerns related to innovative treatments	3: When innovation relied on self-management, patients experienced it as a source of control over symptoms and of personal validation, but also as an additional responsibility. Depending on the availability of support and individual resources, it could generate feelings of isolation, cognitive burden, or anxiety.
**A1.4 (C)** Innovation co-developed with patients is perceived as transferable and empowering.<br>**A2.1 (U)** The MBO programme is perceived as a source of autonomy, security, and symptom control.<br>**A2.4 (U)** The innovation is experienced as a learning process that enables patients to regain control.<br>**A4.1 (U)** The programme provides improved well-being and restored autonomy, helping participants feel physically capable.<br>**A4.6 (U)** Exercise is perceived as promoting well-being and motivating continued engagement.<br>**A13.5 (U)** Walking independently to the operating theatre is experienced as empowering.<br>**A14.5 (U)** Visual Education (VE) supports decisional autonomy when it enables patients to inform themselves and independently weigh options.<br>**A14.6 (U)** Some patients consider decision-making to remain personal and to require active information seeking beyond VE.<br>**A15.1 (U)** Access to a digital platform fosters autonomy and emotional control.<br>**A15.5 (U)** Access to information enables self-advocacy.<br>**A15.6 (U)** Survivors of childhood cancer seek tools to support transition to adult care and strengthen autonomy.<br>**A17.4 (C)** Oral administration involves a mental burden related to daily intake.<br>**A17.5 (C)** Oral anticancer treatment modifies the relationship with the hospital and follow-up care: relief at avoiding hospital visits but a sense of loneliness.<br>**A17.6 (C)** Oral anticancer treatment requires a sometimes constrained form of self-management, with patients managing independently and assuming responsibility for treatment.	4. Providing self-care: between a sense of autonomy and additional burden	
**A5.2 (U)** Immediate priority is survival rather than participation in the RCT.<br>**A5.8 (U)** Lack of evidence on proton therapy generates uncertainty.<br>**A5.9 (U)** Randomisation is experienced as a loss of control.<br>**A7.12 (U)** A physician's recommendation leads to strong adherence.<br>**A9.1 (U)** Some patients actively seek to understand and verify that the proposed treatment is the best option for them.<br>**A9.2 (U)** Some patients describe a passive role and largely follow the oncologist's recommendations.<br>**A9.3 (U)** Some patients consult multiple specialists and travel long distances to access expertise or a recognised centre.<br>**A9.4 (U)** The risk of autoimmune side effects is perceived as disproportionate to expected benefits, leading some patients to refuse access to the innovation.<br>**A9.5 (U)** Some patients rely on family members or close contacts with medical expertise to help them understand therapeutic options.<br>**A9.6 (U)** Some patients actively seek clinical trials and travel specifically to assess eligibility.<br>**A9.7 (U)** Clinical trials are sometimes viewed as a promising option, generating interest.<br>**A9.8 (U)** Some patients maintain a highly optimistic view of their situation despite the perceived severity of the disease.<br>**A9.9 (U)** Hope that treatment is "better than nothing" motivates acceptance of experimental options.<br>**A9.10 (U)** A trusting relationship and good communication with the oncologist are perceived as very important.<br>**A9.11 (U)** Some patients describe shared decision-making with their oncologist.<br>**A9.12 (U)** Some patients describe decision-making with family members when relatives have a medical or advisory role.<br>**A9.13 (U)** Some patients make decisions intuitively, relying on a feeling or inner conviction regarding the place or treatment.<br>**A9.14 (U)** Some patients describe a rational decision-making strategy based on analysis of benefits and risks.<br>**A9.15 (U)** Some patients feel they have "no choice" regarding certain therapeutic options or trials.<br>**A9.16 (U)** The desire to survive and prolong life is a central driver of treatment decisions, even when perceived as frightening.<br>**A9.17 (U)** For some patients, quality of life outweighs therapeutic options perceived as too detrimental to cognitive functioning or identity.<br>**A9.18 (U)** Altruism—the desire to help other patients or future generations—is an important motivation for trial participation.<br>**A10.1 (C)** Participation in the Remote Health Check (RHC) is motivated by a moral logic of giving, with patients wishing to benefit others even without expecting personal benefit.<br>**A13.3 (U)** The decision to participate is based on the promise of rapid recovery, perceived as motivating.<br>**A14.5 (U)** Visual Education (VE) supports decisional autonomy when it enables patients to inform themselves and independently weigh options.<br>**A14.6 (U)** Some patients consider decision-making to remain personal and to require active information seeking beyond VE.<br>**A17.3 (C)** Patients compare oral anticancer treatments with injectable therapies in terms of side effects.<br>**A18.1 (C)** Access to the trial is experienced as a therapeutic opportunity not to be missed, under time pressure.<br>**A18.2 (C)** Being able to reconsider one's decision is reassuring and facilitates choice.<br>**A18.4 (C)** Consent is sometimes signed very quickly, occasionally without full reading, in a context of emotional urgency.<br>**A18.5 (C)** Some patients do not fully perceive the implications of signing consent.<br>**A18.6 (C)** The trial is experienced as the absence of therapeutic alternatives, with a feeling of having "no choice".<br>**A18.7 (C)** The trial is invested with hope, enabling patients to "hold on for others", particularly family members.<br>**A18.8 (C)** Moral conflicts with relatives (partners, physician friends) arise around participation.<br>**A18.10 (C)** The trial is interpreted within a moral logic of giving and reciprocity in support of research.<br>**A18.11 (C)** Trial-related side effects are accepted as the lesser evil, despite uncertainty.<br>**A18.14 (C)** After multiple lines of treatment, some patients feel ready to enter a trial.<br>**A19.1 (U)** Access to targeted therapies is perceived as a temporary chance to prolong life, without illusion of cure.<br>**A19.5 (U)** Targeted therapies are perceived as effective but unstable, with a limited duration over time.	6. Making decisions between therapeutic options	
**A7.1 (U)** The taste of oral nutritional supplements (ONS) is often perceived as unpleasant.<br>**A7.2 (U)** Availability of multiple flavours would facilitate adherence.<br>**A7.3 (U)** Choice of product form is influenced by ease of storage.<br>**A7.4 (U)** Liquid formulations facilitate calculation of nutritional intake.<br>**A7.5 (U)** Gastrointestinal side effects (nausea, diarrhoea, vomiting) hinder adherence.<br>**A7.6 (U)** Patients develop strategies to reduce discomfort when taking ONS.<br>**A7.7 (U)** Cost influences the ability to continue ONS treatment.<br>**A7.8 (U)** Hospitals are perceived as the safest option for obtaining ONS.<br>**A7.9 (U)** Online purchases are cheaper but perceived as less secure.<br>**A7.10 (U)** Preparation of ONS is often perceived as burdensome.<br>**A7.11 (U)** Some patients find ONS easy to use.<br>**A7.13 (U)** Lack of dietary guidance makes ONS use difficult.<br>**A7.21 (U)** Limitations in digital use led some patients to prefer direct channels (e.g., telephone), perceived as more accessible than technological innovations.<br>**A12.6 (U)** The VR headset is perceived as physically uncomfortable due to its weight.	12. Everyday constraints in using healthcare innovations	

**A12.7 (U)** Use of immersive virtual reality (iVR) may be limited by lack of technical familiarity and difficulty handling the equipment.<br>**A12.8 (U)** iVR may cause visual fatigue or ocular discomfort.<br>**A15.9 (U)** Appointment reminders, flexibility, and transport adaptations increase access and engagement.<br>**A15.13 (U)** Digital tools may compromise patient confidentiality when notifications are visible on shared family devices, inadvertently exposing sensitive information.		

Findings	Category	Synthetized findings
**A6.11 (C)** Patients adapt to physical limitations induced by illness and treatments to preserve everyday life.<br>**A6.14 (U)** Treatment is perceived as a therapeutic breakthrough because it mobilises the immune system rather than directly attacking cancer.<br>**A17.1 (C)** Oral treatment alters body image and serves as a daily reminder of the illness.<br>**A17.2 (C)** The disease symbolises chronicity and ongoing uncertainty.<br>**A19.1 (U)** Access to targeted therapies is perceived as a temporary opportunity to prolong life, without illusion of cure.<br>**A19.2 (U)** Patients experience suspended temporality, oscillating between hope and resignation.<br>**A19.3 (U)** Treatment is experienced as ambiguous, between life prolongation and absence of cure.<br>**A19.5 (U)** Targeted therapies are perceived as effective but unstable, with a limited duration over time.<br>**A19.6 (U)** The body becomes dependent on the medication, sometimes experienced as being "enslaved to the treatment".<br>**A19.9 (U)** Precision medicine is accompanied by a sense of existential exhaustion.<br>**A19.10 (U)** Some patients feel isolated due to the rarity of their cancer.<br>**A19.11 (C)** Targeted therapies modify perceptions of the body, being experienced as a biological device that "contains" and controls the cancer.	8. Living with a disease that persists over time	4: Patients sought spaces of respite within the care pathway, allowing them to reconnect with social, cultural, or sensory dimensions that were not centred on the disease.
**A4.2 (U)** The group is experienced as a supportive and motivating community.<br>**A4.3 (U)** Participating in the group helps focus on health rather than illness.<br>**A4.4 (U)** Conventional gyms are perceived as impersonal and unsuitable after cancer.<br>**A4.5 (U)** The fixed structure of the programme and regular sessions create commitment and a sense of responsibility towards the group.<br>**A11.3 (U)** The group is perceived as a safe space to discuss literature rather than illness.<br>**A11.9 (U)** Literary exchanges within the group are experienced as rewarding and supportive.<br>**A11.10 (U)** The programme creates a sense of commitment, framed as a positive obligation to read and listen.<br>**A11.11 (U)** Reading enables patients to reaffirm an identity not reduced to cancer, relegating illness to a less central place.<br>**A15.2 (U)** Connecting with other survivors validates experiences and fosters a sense of being understood.<br>**A15.3 (U)** Social bonds among survivors are experienced as therapeutic and as a source of community beyond illness.<br>**A15.12 (U)** Peer connections can be helpful, but "co-rumination" is perceived as a risk by some.	9. Community support and shared experiences	
**A4.1 (U)** The programme provides improved well-being and restored autonomy, helping participants feel physically capable.<br>**A11.1 (U)** Patients are motivated by the idea of rediscovering the pleasure of reading and reconnecting with a valued intellectual activity.<br>**A11.2 (U)** Fatigue, lack of motivation, and distractions limit physical activity, with audiobooks emerging as a more accessible alternative.<br>**A11.4 (U)** Audiobooks carry strong emotional meaning and provide emotional support.<br>**A11.5 (U)** Audio listening facilitates concentration by setting the pace of walking.<br>**A11.6 (U)** Audiobooks offer a guided immersive experience that alters how patients perceive their care journey.<br>**A11.7 (U)** Audiobooks help avoid the feeling of "wasting time" associated with traditional reading.<br>**A11.8 (U)** Audiobooks promote falling asleep and provide nighttime soothing, reducing bedtime-related anxiety.<br>**A11.12 (U)** Some literary content triggers discomfort when addressing darker themes.<br>**A11.13 (U)** The intervention enables a rediscovery of the cultural pleasure of reading.<br>**A11.14 (U)** Books stimulate imagination and creativity.<br>**A11.15 (U)** The programme reinforces the notion of "taking care of oneself".<br>**A11.16 (U)** Activities outside clinical care (e.g., walking) provide relaxation, bodily awareness, and a sense of acting positively for oneself.<br>**A11.17 (U)** The combination of reading and walking benefits well-being and the perception of self-care.<br>**A11.18 (U)** Outdoor activity provides relief and helps manage anxiety.<br>**A12.1 (U)** Immersive virtual reality (iVR) is experienced as a discovery and sense of wonder, allowing exploration of unfamiliar places.<br>**A12.2 (U)** Visual immersion generates positive emotions related to colours, shapes, and the beauty of the virtual environment.<br>**A12.3 (U)** iVR helps reduce anxiety during chemotherapy by providing complete distraction.<br>**A12.4 (U)** Use of iVR alters time perception, making treatment sessions feel shorter.<br>**A12.5 (C)** The device provides sensory immersion but requires adequate sound isolation to be effective.	10. Moments of distraction and pleasure throughout the care pathway	
**A6.7 (C)** A sense of abandonment.<br>**A13.10 (U)** Patients feel confident when they believe that care teams would not allow discharge if there were any risk.<br>**A16.5 (U)** The pathway prior to accessing the innovative service is described as confusing and stressful, marked by delays, diagnostic errors, and poor communication.<br>**A18.9 (C)** The trial reinforces a sense of being recognised, supported, and surrounded within the research unit.<br>**A18.12 (C)** The trial repairs a previously experienced sense of abandonment within the healthcare system.<br>**A20.1 (U)** RT Prepare (Radiotherapy Prepare Program) is perceived as a privileged space for listening, allowing patients to express concerns and feel reassured before radiotherapy.<br>**A20.6 (C)** A calm, private, and appropriate environment (e.g., a room with a window) enhances feelings of reassurance.	13. Trust and disruption in the relationship with the healthcare system	

Web Appendix Figure 2: Practice, policy, and research recommendations derived from the synthesis

RECOMMENDATIONS FOR PRACTICE

Recommendation 1: Supporting access to innovation through progressive and accessible information beyond the hospital setting.

Population: Adults living with cancer who are exposed to a health innovation.

Phenomenon of interest: Access to innovation through understanding of information.

Context: Oncology care pathways.

Recommendation: For adult patients with cancer, healthcare teams should distribute information related to innovation across multiple points in the care pathway (e.g., announcement, decision-making, initiation, and follow-up) and provide materials that can be consulted later (e.g., written documents, educational videos, digital platforms) (18). Understanding may be revisited and checked at different moments, without assuming that a single exchange is sufficient, while considering variations in patients' availability reported in the studies (e.g., fatigue, pain, treatment-related side-effects) (16,21).

Justification: Across the included studies, patients described understanding innovation when they were able to revisit information at times when they felt cognitively and emotionally available, rather than during single or time-limited exposures. This finding was reported consistently across multiple studies, supporting a moderate level of confidence in this recommendation.

Informed by: Synthesised Finding 1

JBI Grade: B

Recommendation 2: Ensuring relational continuity in access to innovation.

Population: Patients engaged in an innovative care pathway.

Phenomenon of interest: Relational continuity perceived as facilitating access to innovation.

Context: Clinical trials, radiotherapy, targeted therapies, and chronic cancer treatments.

Recommendation: For patients engaged in innovative care pathways, healthcare services should ensure that each patient has an identified point of contact regarding the innovation (e.g., a referring oncologist, advanced practice nurse, or clinical research coordinator), with clear modalities for contact (e.g., scheduled consultations, dedicated phone number, secure messaging) (16,25). Limiting changes in interlocutors may contribute to reducing discontinuities in care.

Justification: Across the included studies, patients described access to innovation as being facilitated when it was supported by identified and consistent care professionals. Conversely, changes in interlocutors were reported as generating confusion, uncertainty, or disengagement from care. These findings were reported across multiple studies, supporting a moderate level of confidence in this recommendation.

Informed by: Synthesised Finding 2

JBI Grade: B

Recommendation 3: Clarifying care components associated with self-management.

Population: Patients undertaking treatment or monitoring-related care at home.

Phenomenon of interest: Perceived self-management.

Context: Oral treatments, remote monitoring, digital tools, and educational programmes.

Recommendation: For patients engaged in self-management, healthcare teams should explicitly outline the expected tasks (e.g., medication intake, symptom monitoring, alert thresholds), provide materials that can be consulted later (e.g., alert sheets, tutorials), and clarify modalities for feedback and contact with care teams (e.g.,

contact points, response times, communication channels) (17,24). This clarification should be provided before the initiation of self-management and revisited during follow-up to account for difficulties reported by patients.

Justification: Across the included studies, patients described self-management as being facilitated when it was supported by explicit guidance and accessible points of contact. In the absence of such a framework, self-management could be experienced as a difficult transfer of responsibility.

Informed by: Synthesised Finding 3

JBI Grade: B

Recommendation 4: Integrating non-pharmacological supportive innovations as components of the care pathway.

Population: Patients receiving oncology care.

Phenomenon of interest: Experiences of respite within spaces not centred on the disease.

Context: Palliative care, supportive care, and survivorship settings.

Recommendation: Healthcare services should develop and facilitate access to spaces of respite (e.g., peer support groups, creative workshops, cultural activities, immersive interventions) and integrate them within care pathways as complementary components supporting quality of life and the continuity of technical care (17).

Justification: Across the included studies, patients described these spaces as moments in which illness and technical care were no longer in the foreground, providing periods experienced as relief or respite within the care trajectory.

Informed by: Synthesised Finding 4

JBI Grade: B

POLICY RECOMMENDATIONS

Policy Recommendation P1: Ensuring institutional stability of coordination roles within innovative care pathways.

Recommendation: Health-care organisations and decision-makers are encouraged to ensure the institutional stability of coordination functions within innovative care pathways, by maintaining identified points of contact and clear modalities of communication for patients (e.g., dedicated phone lines, secure messaging systems, scheduled consultations) (24).

Justification: Across the included studies, patients reported that access to innovation was facilitated when coordinating professionals remained identifiable and stable over time. Conversely, organisational discontinuities or changes in interlocutors were described as generating confusion, uncertainty, or disengagement from care.

Informed by: Synthesised Finding 2

Policy Recommendation P2: Improving the visibility of material and organisational steps required to access innovation.

Recommendation: Healthcare organisations may clarify logistical and organisational elements related to access to innovation (e.g., contact details, locations, timelines, schedules, and points of contact) and integrate this information into admission and follow-up processes (e.g., information documents, digital platforms, physical supports) (18,25).

Justification: Across the included studies, patients described access to innovation primarily through organisational and temporal reference points rather than through technical characteristics of the innovation itself. Clear and visible logistical information supported their ability to navigate and engage with innovative care pathways.

Informed by: Synthesised Finding 2

Policy Recommendation P3: Integrating spaces of respite within oncology care pathways.

Recommendation: Healthcare organisations and partner networks may support the development of spaces of respite (e.g., creative, community-based, or immersive interventions) and integrate them into proposed care pathways, ensuring their accessibility and sustainability, while avoiding their presentation as therapeutic alternatives to biomedical care.

Justification: Across the included studies, patients described these spaces as enabling a temporary distancing from illness and technical care, providing periods experienced as relief or respite within the overall care trajectory.

Informed by: Synthesised Finding 4

RESEARCH RECOMMENDATION

Research Recommendation R1: Exploring under-represented contexts of access to innovation.

Future research should document experiences of access to innovation in contexts that remain under-represented in the literature, including patients living in rural areas, individuals from linguistic or cultural minority groups, patients with multimorbidity, and those receiving care outside expert or specialised centres (24). Qualitative longitudinal or ethnographic studies would be particularly relevant to deepen understanding of these contexts, which are currently insufficiently described.

Informed by: Synthesised Findings 1, 2, and 4

Research Recommendation R2: Investigating how self-management is experienced by patients.

Qualitative studies should explore how patients perform, and experience care delivered autonomously outside the hospital setting, the types of support they rely on (e.g., health-care professionals, informational tools, relatives), and the circumstances in which they report difficulties or barriers (24). Participatory approaches centred on patient experience could further enrich this line of research.

Informed by: Synthesised Finding 3
